# Biological and Phytochemical Profile of Volatile Oils from Romanian Mountain Flora

**DOI:** 10.3390/ph19081293

**Published:** 2026-08-15

**Authors:** Lidia-Ioana Virchea, Maria Totan, Cecilia Georgescu, Adina Frum, Endre Máthé, Monica Mironescu, Bence Pecsenye, Robert Nagy, Oana Viorica Danci, Maria-Lucia Mureșan, Nastaca Alina Palade, Felicia-Gabriela Gligor

**Affiliations:** 1 Faculty of Medicine, “Lucian Blaga” University of Sibiu, Lucian Blaga Str. 2A, 550169 Sibiu, Romania; lidia.virchea@ulbsibiu.ro (L.-I.V.); adina.frum@ulbsibiu.ro (A.F.); maria.muresan@ulbsibiu.ro (M.-L.M.); nastacaalina.coman@ulbsibiu.ro (N.A.P.); felicia.gligor@ulbsibiu.ro (F.-G.G.); 2 Faculty of Agriculture Sciences, Food Industry and Environmental Protection, “Lucian Blaga” University of Sibiu, Dr. Ion Rațiu Str. 7-9, 550012 Sibiu, Romania; monica.mironescu@ulbsibiu.ro; 3 Institute of Nutrition Science, Faculty of Agricultural and Food Sciences and Environmental Management, University of Debrecen, Böszörményi Str. 128, 4032 Debrecen, Hungary; endre.mathe@agr.unideb.hu (E.M.); pecsenye.bence@agr.unideb.hu (B.P.); nagy.robert@agr.unideb.hu (R.N.); 4 Department of Life Sciences, Faculty of Medicine, Vasile Goldis Western University of Arad, L. Rebreanu Str. 86, 310414 Arad, Romania; 5 Faculty of Sciences, “Lucian Blaga” University of Sibiu, Dr. Ion Rațiu Str. 5-7, 550012 Sibiu, Romania; oana.danci@ulbsibiu.ro

**Keywords:** essential oils, phytochemicals, polyphenols, natural antioxidants, antimicrobial effect, *Drosophila melanogaster*, toxicity

## Abstract

**Background/Objectives:** Essential oils (EOs) are complex mixtures extracted from plants. The aim of this study was to evaluate the chemical composition, antioxidant and antimicrobial properties, as well as in vivo toxicity against *Drosophila melanogaster* of the EOs extracted from *Achillea millefolium* L. (AM), *Mentha longifolia* L. (ML), and *Thymus serpyllum* L. (TS) from the mountains in Sibiu County, Romania. **Methods:** EOs were extracted by hydrodistillation. Their phytocompounds were determined by Gas Chromatography–Mass Spectrometry. Antioxidant potential was evaluated by 2,2-diphenyl-1-picrylhydrazyl (DPPH), 2,2′-azinobis [3-ethylbenzothiazoline-6-sulfonic acid]-diammonium salt (ABTS), and ferric-reducing antioxidant power (FRAP) assays. Antibacterial action was measured against eight pathogenic bacteria. An in vivo *D. melanogaster* diet-dependent viability assay was conducted to assess the toxicity of the EOs. **Results:** Chamazulene (49.72%), pulegone (36.20%), and thymol (42.11%) were the most abundant constituents of the AM, ML, and TS EOs, respectively. All tested EOs exerted antioxidant activity, with TS EO being the most efficient in the DPPH (98.19% inhibition) and ABTS (1.82 mmol Trolox equivalent (TE)/mL EO) scavenging assays, while AM EO performed best in the FRAP assay (66.70 μmol TE/mL EO). Against TS EO, *Bacillus subtilis* was the most sensitive, with an inhibition zone of 37 mm. A dose-dependent decrease in the viability of *D. melanogaster* L. was observed when their diet was supplemented with AM, ML, and TS EOs. **Conclusions**: The analyzed EOs demonstrated antioxidant and antimicrobial effects, suggesting their potential applications in various fields. However, due to their toxicity, an in-depth analysis is required before introducing such products in therapy.

## 1. Introduction

Essential oils (EOs), also known as volatile oils, have started to be highly demanded products, with their market experiencing significant growth in recent years globally, with a market size of USD 25.86 billion in 2024 and a forecast to reach USD 56.25 billion by 2033 [1]. EOs have various applications in food, agriculture and cosmetic industries [2]. Moreover, based on their therapeutic effects, essential oils and their bioactive compounds are intensively studied for the development of new pharmaceutical products [3]. This increased demand is a result of people’s awareness of the health benefits of natural products, including EOs [4].

EOs have a complex composition and a characteristic smell [5]; generally, they contain two or three major constituents, along with other minor constituents, reaching a total of 20 to 60, and sometimes over 300, different compounds. EOs consists of terpenes, terpenoids, and phenylpropanoids, with mono- and sesquiterpenes being the predominant classes [6]. Various conventional and advanced extraction methods were previously carried out to obtain essential oils from plant materials, with hydrodistillation being the oldest one; it is a simple procedure that uses the Clevenger apparatus. In this method, the plant material is immersed in water in a round-bottom flask and boiled. The volatile compounds pass through a condenser and then enter a separator, where the volatile oil is separated from the water. This method is still applied nowadays for essential oil extraction, even if other advanced techniques, with higher extraction yields, lower costs, and reduced extraction time, are available, such as supercritical fluid extraction or solvent-free microwave extraction [7,8].

EOs are well-known for their antioxidant, antimicrobial, anticancer, and anti-inflammatory properties [9]. Biological effects are mainly attributed to predominant components, but even minor components can influence biological activity by additive or antagonistic effects [6]. Due especially to their phenolic content, EOs are involved in combating oxidative stress, which is one of the causes of neurodegenerative diseases [10]. Moreover, in the context of increasing bacterial resistance to antibiotics, previous studies have demonstrated that, due to their antibacterial effect, essential oils could be a suitable alternative therapy for infections with resistant pathogens [11].

EOs and their constituents are generally considered safe when used in adequate amounts. However, some monoterpenes are harmful, especially in high doses [12]. Previous studies have already assessed the toxicity of volatile oils and plant extracts against a *Drosophila melanogaster* model system [13,14,15,16,17]. *Drosophila melanogaster* (fruit fly) is a versatile organism used for more than 100 years in science, having about 75% of its disease-related genes similar to humans, which presents many advantages compared to vertebrates. It has a short life cycle with four stages (egg, larva, pupa, and fly) and a high reproductive rate; it is also cost-effective and has fewer ethical and safety restrictions [18].

*Achillea millefolium* L. (yarrow) is a medicinal plant species belonging to the Asteraceae family. It has a long history of use in European traditional medicine with respect to alleviating bleedings, inflammations, and gastrointestinal and skin disorders [19]. This plant contains essential oils (including chamazulene [20,21,22,23], β-pinene [20,21,24], caryophyllene [21,23], gremacrene-D [20,23], 1,8-cineole [20,21,22,24,25,26,27,28], sabinene [21,25,26], camphor [22,24,26,27], and borneol [22,28,29] as major constituents) and polyphenolic compounds [20]. Some pharmacological effects of *A. millefolium* L. essential oils, including antioxidant [24,25,30] and antimicrobial [24,27,29] properties, have been demonstrated.

*Mentha longifolia* L. (wild mint) is a medicinal and aromatic plant from the Lamiaceae family. It is known in traditional medicine for ameliorating respiratory, digestive, and skin conditions [31]. *M. longifolia* contains essential oils (with pulegone [32,33,34], piperitone oxide [34,35], β-caryophyllene [33], (–)-germacrene D [36], 1,8-cineole [32], 1-menthone, camphor [34], and carvone [37] among dominant components), phenolic compounds, phytosterols, and fatty acids [31]. Some of the biological effects of *M. longifolia* L. essential oil have been reported in the literature, such as antioxidant [36,37], antimicrobial [36,37,38], anti-inflammatory, and antispasmodic effects [31].

*Thymus serpyllum* L. (wild thyme) is a perennial medicinal shrub from the Lamiaceae family. In traditional medicine, wild thyme is used to alleviate gastrointestinal and respiratory ailments [39,40,41]. *T. serpyllum* L. contains volatile oils (rich in thymol [42,43,44,45,46], carvacrol [47,48], methyl carvacrol [47], and 1,8-cineole [48], among other constituents), phenol carboxylic acids, and flavonoids [39,40,41]. *T. serpyllum* L. volatile oil has antimicrobial [27,49,50,51,52], antioxidant [43,49,50], and anticancer [50,53] effects.

To the best of our knowledge, there are few studies in the literature that have investigated the chemical composition and antimicrobial and antioxidant activities of the volatile oils extracted from these three species, harvested from Romania. Furthermore, no previous studies have assessed the in vivo toxicity of *A. millefolium* L., *M. longifolia* L., and *T. serpyllum* L. essential oils against *Drosophila melanogaster*. The aim of this study was to analyze the chemical composition, antioxidant and antibacterial activities, as well as toxicity against *Drosophila melanogaster* of the volatile oils extracted from *Achillea millefolium* L., *Mentha longifolia* L., and *Thymus serpyllum* L. collected from the mountainous wild flora of Sibiu County, Romania.

## 2. Results and Discussion

### 2.1. Volatile Oil Extraction Yields and Organoleptic Analysis

Volatile oils were extracted by hydrodistillation from the aerial parts of *A. millefolium* L., *M. longifolia* L., and *T. serpyllum* L. The mean values of extraction yields and organoleptic properties of the essential oils analyzed in this study are presented in Table 1. Extraction yields varied by plant species, with *M. longifolia* L. containing the highest amount of volatile oil, followed by *T. serpyllum* L. and *A. millefolium* L.

The results are in accordance with the provisions of the European Pharmacopoeia, 10th Edition, for *T. serpyllum* L. and *A. millefolium* L., which stipulates a minimum content of 3 mL/kg for *T. serpyllum* L. essential oil extracted from dried flowering aerial parts (*Serpylli herba*) and 2 mL/kg for *A. millefolium* L. essential oil extracted from dried flowering tops (*Millefolii herba*) [54,55]. Also, the extraction yield is in accordance with the Romanian Pharmacopoeia, Xth Edition, which stipulates a minimum content of 0.2%, *m*/*v*, for *A. millefolium* L. inflorescences (*Millefolii flos*) [56].

The volatile oil content of *A. millefolium* L. obtained in the present study is consistent with the findings of other researchers, who reported a volatile oil content of between 0.068 and 1.16% in various plant parts collected from different geographical areas [23,26,27,28,29,30,57,58,59,60]. Other studies reported similar volatile oil content in *A. millefolium* L. collected from Sălaj County, Romania (dry inflorescences) [20], Iran (aerial part at flowering stage) [22], India (fresh flowers collected at an altitude of 1700 m) [25], Saudi Arabia (leaves) [57], and Samara region, Russia (full flowering herb) [21]. Higher content was reported in *A. millefolium* L. from the Apuseni Mountains and Western Plain of Romania (vegetal parts) [58] and Saudi Arabia (aerial parts and stems) [57], while *A. millefolium* L. from the hill area of Romania (fresh flowers) [59], Canada (flowers and leaves) [60], Bosnia and Herzegovina (full flowering stage) [30], Serbia (aerial parts) [27,29], Dagestan (aerial parts) [28], and the United States of America (inflorescences) [26] had a lower essential oil content. In accordance with our observations, other researchers reported a dark blue color for *A. millefolium* L. volatile oil due to its high chamazulene content [20], while others observed a yellow color [25,28,30].

The volatile oil content of *M. longifolia* L. reported in the scientific literature varied between 0.20 and 3.6% [33,34,35,36,61,62], which is in accordance with the results of the present study. Higher essential oil concentrations were found in *M. longifolia* L. collected from Egypt [61] and South Algeria [62], while *M. longifolia* L. from Morocco [36], Saudi Arabia [33], Turkey [34], and Pakistan [35] had lower volatile oil content. Other researchers observed similar organoleptic properties for *M. longifolia* L. volatile oil [62].

*T. serpyllum* L. volatile oil content obtained in the present study is in accordance with the findings of other studies, which reported extraction yields between 0.37 and 0.9% [47,63,64]. As we expected, the volatile oil content obtained in this study is consistent with that reported for *T. serpyllum* L. collected from different regions of Transylvania, which varied between 0.38 and 0.50% [47]. Higher extractions yields were obtained for *T. serpyllum* L. collected from different Carpathians regions in Ukraine, which varied according to microclimatic conditions [63].

Apart from the plant harvesting area, the plant part and humidity could also impact the extraction yield. Higher volatile oil content was found in stems of *A. millefolium* L. than in roots, leaves, and flowers [58]. The volatile oil content extracted from fresh flowers of *A. millefolium* L. collected from the hill area of Romania was only 0.15% [59]. Also, the extraction yield was lower (0.20%) for fresh aerial parts of *M. longifolia* L. from Saudi Arabia [33] than the volatile oil content in dried aerial parts investigated in the present study.

### 2.2. Chemical Composition of the Volatile Oils

Gas Chromatography–Mass Spectrometry (GC-MS) analysis was performed to establish the chemical composition of the studied essential oil. Twenty compounds were identified in the volatile oil of *A. millefolium* L., representing 99.92% of the total volatile components. The GC-MS chromatogram can be found in the Supplementary Materials (Figure S1). Chamazulene (49.72%) was the most abundant constituent. Other predominant compounds, with a concentration over 4%, were: eucalyptol (or 1,8-cineole), β-pinene, germacrene D, sabinene, and caryophyllene. Some volatile constituents were identified in lower concentrations (Table 2).

The composition of the volatile oil of *A. millefolium* L. is influenced by the plant’s ploidy level. Diploid and tetraploid plants contain mainly proazulenes which are converted to chamazulene during the heating process. Moreover, tetraploid plants contain α-pinene, β-pinene, and cariophyllene. In hexaploid plants, camphor, sabinene, 1,8-cineol, and β-pinene are the main constituents, but these types do not contain azulene sesquiterpenes. In octaploid plants, linalool is the major constituent [65]. Based on the above-mentioned information, we identified the *A. millefolium* L. investigated in this study as a tetraploid species.

A high amount of chamazulene is a feature of essential oil extracted from *A. millefolium* L. harvested from Romania. Its concentration varies by the part of the plant used for extraction, plant harvesting area and period of harvesting [20,23,58]. Previous studies reported that yarrow stems collected from the Apuseni Mountains and Western Plain of Romania contained a high amount of chamazulene (45.79%), followed by roots (33.82%) collected in the same area [58], and inflorescences (25.26%) collected from Sălaj County, Romania [20]. Regarding harvesting period, other researchers found that in 2022, the chamazulene content of *A. millefolium* L. collected from Mureș County, Romania, was higher (25.37%) than in 2021 (21.97%) [23]. However, chamazulene was absent in some *A. millefolium* L. essential oils [59]. A previous review highlighted that chamazulene is a bicyclic hydrocarbon derived from azulene, with antibacterial, antiallergic, and anti-inflammatory effects [66].

The results are consistent with those of another study that reported β-pinene (32.11%), trans-caryophyllene (9.15%), eucalyptol (6.58%), and germacrene D (5.59%) as the main constituents of *A. millefolium* L. volatile oil [20]. In contrast with our findings, other studies reported higher germacrene D, caryophyllene [23], camphor, and eucalyptol in the volatile oil from *A. millefolium* L., while β-pinene, germacrene D, sabinene, and caryophyllene were not reported [59]. According to a previous review, eucalyptol, also called 1,8-cineole, has anti-hypertensive, antimicrobial, anti-inflammatory, and anti-nociceptive effects. It demonstrated valuable properties in respiratory disorders, such as asthma and chronic obstructive pulmonary disease [67]. β-pinene in combination with paclitaxel proved to have a synergistic cytotoxic effect against non-small cell lung cancer cell lines [68]. β-pinene, germacrene D, and β-caryophyllene exhibit antimicrobial and antioxidant activities [69].

The volatile oil of *A. millefolium* L. collected from Turkey had a different composition, with the following major constituents: 1,8-cineole (75.19%), α-phellandrene (5.53%), P-eugenol (5.53%), camphor (5.45%), α-terpineol (2.09%), β-pinene (1.66%), camphene (1.20%), and α-pinene (1.02%) [24]. Monoterpenoids such as 1,8-cineole, (–)-δ-cadinol, camphor, α-thujone, and borneol predominated in the composition of the oil extracted from *A. millefolium* L. aerial parts collected from Dagestan [28], while oxygenated sesquiterpenes, with elemol being the major component of this oil, predominated in the volatile oil of *A. millefolium* L. collected from Bosnia and Herzegovina [30].

Twenty-two compounds were identified in the volatile oil of *Mentha longifolia* L., representing 99.33% of the total volatile components. The GC-MS chromatogram can be found in the Supplementary Materials (Figure S2). Pulegone (36.20%) was the most abundant constituent. Other major compounds were cis-menthone (17.89%), caryophyllene (9.56%), piperitone oxide (9.21%), germacrene D (5.29%), isomenthone (4.52%) and 2,6-dimethyl hydroquinone (4.10%). Some volatile constituents were identified in lower concentrations (Table 3).

The results are aligned with those of other studies in the literature, which reported high pulegone content in the essential oil of *M. longifolia* L. harvested from Iran (49.20%) [70], South Algeria (31.85%) [62], and Egypt (28.60%) [61].The essential oil of *M. longifolia* L. collected from Saudi Arabia contained higher pulegone content (82.02%) [33], while its content was lower in the essential oil of *M. longifolia* L. harvested from other habitats in Iran (17.53%) [70], Turkey (12.47%) [34], and Pakistan (12.20%) [35]. Pulegone is a monoterpene ketone, isolated for the first time from *Mentha pulegium* L. This compound exerts various biological effects, including antimicrobial, antipyretic, and antihistaminic effects [71].

Studies that have investigated the chemical composition of the volatile oil of *M. longifolia* L. harvested from Romania are limited. Higher piperitone oxide (36.74%) and β-myrcene (7.38%) content was found in the aerial parts of *M. longifolia* L. collected in the vegetative stage from Vaslui County, Romania [72]. Previous studies reported greater piperitone oxide content in the volatile oil of *M. longifolia* L. collected from Morocco (53.43%) [36], Pakistan (33.90%) [35], and Turkey (15.55%) [34].

Higher amounts of caryophyllene (20.02%) and (–) germacrene D (16.53%) were found in the volatile oil of *M. longifolia* L. collected from Morocco [36]. Eucalyptol content varies widely in the volatile oil of *M. longifolia* L., ranging between 2.41 and 24.4% [61,70,73]. Almost identical eucalyptol content was reported in the volatile oil of *M. longifolia* collected from South Algeria (5.72%) [62] and Turkey (5.73%) [34]. Some studies reported greater amounts of isomenthone in *M. longifolia* L. volatile oil [61,62]. Other volatile constituents from *M. longifolia* L. volatile oil were quantified, including menthone, piperitenone, piperitenone oxide [70], carvone [62], and camphor [34].

Twenty-one compounds were identified in the volatile oil of *Thymus serpyllum* L., representing 99.61% of the total volatile components. The GC-MS chromatogram can be found in the Supplementary Materials (Figure S3). Thymol (42.11%) was the most abundant compound. Other predominant compounds were p-cymene (16.34%), γ-terpinene (10.97%), carvacrol (8.71%), methyl carvacrol (6.03%) and methyl thymol (4.45%). Some volatile constituents were identified in lower concentrations (Table 4).

The results are consistent with the findings of other studies in the literature, which reported thymol as the main volatile compound in the essential oil of *T. serpyllum* L. harvested from Turkey (41.03%) [42] and *T. serpyllum* L. with a European origin (47.31%) [43]. Previous studies reported higher thymol content (52.56%) in the volatile oil of *T. serpyllum* L. collected from Ukraine [46] and lower thymol content (10.54%) in the volatile oil of *T. serpyllum* L. from Morocco [49]. Thymol is a natural compound that has been highly researched as a result of its anti-inflammatory and wound healing effects. Due to its anti-inflammatory activity, the compound could be suitable for the improvement of many respiratory, digestive, cardiovascular, and skin disorders [74]. Among other effects, the antioxidant and antimicrobial properties of thymol are well documented [75].

Despite our results, other researchers observed that *T. serpyllum* L. harvested from different Transylvanian regions contained higher amounts of carvacrol than thymol [47,48]. Also, greater carvacrol amounts were reported in the essential oil of *T. serpyllum* L. from Turkey (33.59%) [42] and Algeria (66.00%) [76]. Carvacrol exerts numerous health benefits, including antioxidant, antimicrobial, anti-inflammatory, anticancer, antidiabetic, and cardio-/hepato-/neuro-protective effects [77].

Contrary to those mentioned above, in some *T. serpyllum* L. volatile oils, thymol and carvacrol were not major compounds; geraniol (63.40%) and nerol (18.90%) were the main volatile constituents in the essential oil of *T. serpyllum* L. collected from Serbia. The authors concluded that *T. serpyllum* L. investigated in their study belonged to the geraniol chemotype [27]. Thus, the plant’s growing location influences the composition of its volatile oil.

Similar p-cymene content (18.97%) was detected in the volatile oil extracted from *T. serpyllum* L. collected from the Năsăud area (Bistrița-Nasaud County, Romania), but lower p-cymene content was detected in the essential oil of *T. serpyllum* L. from the Mociu area (Cluj County, Romania) and Tămășesti area (Maramureș County, Romania) [47]. Numerous pharmacological effects of p-cymene have been demonstrated, such as antioxidant, antimicrobial, anti-inflammatory, analgesic, anticancer, and antidiabetic effects [78]. The concentration of γ-terpinene was lower in the samples from Mociu, Tămășești and Năsăud areas. Similar caryophyllene (3.17–4.83%) and higher methyl carvacrol (8.81–13.35%) and β-bysabolene (3.42–5.18) amounts were reported [47]. Recently, γ-terpinene was reported to ameliorate neuropathic pain of tumor origin in an animal model [79]. Compared to our results, the volatile oil of *T. serpyllum* L. collected from the Medicinal Plant Garden of the University of Medicine and Pharmacy in Târgu Mureș, Romania, had higher amounts of p-cymene (25.00%), 1,8-cineole (9.50%), and methyl carvacrolether (11.10%), but lower γ-terpinene (0.30%) and methyl thymolether (0.20%) content [48].

The chemical composition of volatile oils is highly influenced by plant harvesting time, storage conditions and duration [80], plant organ [57], extraction method [57,81], pre-extraction treatment [82], genetic factors [65], and geographic origin of the plant [47]. Differences in the composition of volatile oils will inevitably influence their biological effects [57,81].

The high chamazulene, pulegone, and thymol content of the analyzed volatile oils led to concerns regarding the safety of these products. Therefore, an analysis of the literature showed that thymol, even if it is generally considered as a safe compound, may exhibit toxic effects [83]. It was implicated in dermatitis and allergic reactions. To avoid its toxicity, when used orally, thyme is recommended in a daily dose of lower than 10 g of dried leaves with a thymol concentration of 0.03% [84].

A major concern regarding the toxicity of pulegone is a carcinogenic risk that was observed in animal model studies. This compound was reported to cause urinary bladder cancer in female rats and liver cancer in mice of both sexes. Also, pulegone consumption might lead to bone cancer in female mice [85]. The toxic effect may be attributed to the metabolites of pulegone that occur after oral administration, such as menthofuran and 8-pulegone aldehyde, which are more toxic than pulegone [12]. However, a previous study revealed that pulegone is more toxic than menthofuran in human liver slices [86].

Regarding chamazulene, an in silico study predicted that although it is capable of crossing the blood–brain barrier, its toxicity with respect to different organs, including liver, kidney, heart, and respiratory tract, was not revealed. Moreover, no toxic immunological, mutagenic, or cytotoxic effects were predicted [87].

### 2.3. Antioxidant Capacity

The present study investigated the antioxidant potential of three essential oils extracted from the dried aerial parts of *A. millefolium* L., *M. longifolia* L., and *T. serpyllum* by 2,2-diphenyl-1-picrylhydrazyl (DPPH), 2,2′-azinobis [3-ethylbenzothiazoline-6-sulfonic acid]-diammonium salt (ABTS), and ferric-reducing antioxidant power (FRAP) methods. As we expected, the antioxidant capacity ranking of volatile oils varies depending on the test performed. Results demonstrated that *T. serpyllum* L. essential oil exerted the strongest DPPH free radical scavenging potential, followed by *M. longifolia* L. and *A. millefolium* L. volatile oils (Table 5). All three tested volatile oils possess lower DPPH inhibition activity compared to the ascorbic acid used as a standard antioxidant compound. Similar to the DPPH assay, *T. serpyllum* L. volatile oil was found to be the most effective in the ABTS test, followed by *A. millefolium* L. volatile oil. *M. longifolia* L. essential oil demonstrated a very low antioxidant activity in the ABTS assay. In the case of the FRAP assay, *A. millefolium* L. volatile oil demonstrated the highest antioxidant capacity, followed by *T. serpyllum* L. and *M. longifolia* L. essential oils.

Different rankings of the antioxidant activities of volatile oils tested by DPPH, ABTS and FRAP assays can be attributed to the different mechanisms of each test. Specifically, both DPPH and ABTS methods are based on free radical scavenging. In these tests, the antioxidant effect can be produced by electron transfer and/or hydrogen atom transfer [88,89]. The FRAP assay is based on the reduction of Fe^3+^ to Fe^2+^, in acidic solution, at a pH of 3.6 [90] to facilitate iron dissolution. In the FRAP assay only electron transfer is implicated. Due to the fact that the FRAP assay does not involve hydrogen atom transfer, sometimes this method is not well correlated with other antioxidant tests. To investigate the antioxidant mechanism of a compound, it is recommended to conduct a FRAP assay along with other tests [91]. Different antioxidant compounds can be tested in the above mentioned tests, based on their polarity. Thus, lipophilic compounds are generally more active in DPPH assays because the DPPH radical is soluble in organic polar solvents [89]. In the case of ABTS tests, both hydrophilic and lipophilic compounds react [92]. Only hydrophilic antioxidants react in FRAP assays [89].

The higher DPPH and ABTS free radical scavenging activity of *T. serpyllym* L. volatile oil observed in our study can be attributed to high amounts of phenolic compounds, thymol and carvacrol. Phenolic compounds are well-known bioactive compounds that react, especially in DPPH assays [88]. Previous studies reported that thymol and carvacrol inhibited DPPH free radicals in a dose-dependent manner and suggested that this was a result of the hydrogen atom transfer mechanism [93,94]. Al-Mansori et al. (2020) observed that carvacrol was more effective in ABTS free radical scavenging as compared with thymol [94]. The 1:1 mixture of thymol and carvacrol demonstrated a higher ABTS scavenging activity as compared with thymol alone. Rúa et al. (2019) reported that both thymol and carvacrol inhibited ABTS radicals in a dose-dependent manner [95]. Furthermore, they observed that the combination of these compounds at lower concentrations exerted an additive effect, while mixtures in which both or only one of the components is in a higher concentration demonstrated an antagonistic effect. Other studies also reported the reducing power of thymol and carvacrol [93,94].

The results are consistent with those of another study that demonstrated strong DPPH radical scavenging activity for the volatile oil of *T. serpyllum* L. collected from different locations in Transylvania [47]. Our findings are aligned with the results of previous studies which reported that *T. serpyllum* L. volatile oil, with thymol as the main constituent, inhibited the DPPH free radical [96,97]. Ouedrhiri et al. (2021) reported strong DPPH inhibition for the volatile oil of *T. serpyllum* L., with p-cymene, γ-terpinene, and thymol as major constituents [98]. Salaria et al. (2023) and Al-Mijalli et al. (2025) reported that *T. serpyllum* L. volatile oils exerted scavenging activity against both DPPH and ABTS radicals, as well as a reducing effect demonstrated by a FRAP assay [49,99]. Kulisic et al. (2005) found that *T. serpyllum* L. volatile oil, at 2 g/L, inhibited the DPPH free radical by 82% [100]. Interestingly, only the polar fraction of this oil, in which carvacrol and thymol were the main constituents, exerted an antioxidant effect against the DPPH free radical [100]. These findings support the idea that DPPH radical scavenging activity is attributed to hydroxyl groups (– OH) from the chemical structure of the compounds existing in the volatile oil. Similar to our results, Kulisic et al. (2005) demonstrated that *T. serpyllum* L. volatile oil had a lower DPPH radical scavenging activity than ascorbic acid. Hussain et al. (2013) observed that *T. serpyllum* L. volatile oil, in which the main constituents were carvacrol, *o*-cymene, α-terpineol, α-pinene and β-caryophyllene, inhibited the DPPH free radical more effectively than carvacrol alone [100,101].

The high chamazulene content of *A. millefolium* L. volatile oil could explain the low DPPH free radical scavenging effect and high reducing effect observed for this essential oil. A previous study conducted by Capuzzo et al. (2014) demonstrated that chamazulene isolated from chamomile essential oil has a strong reducing effect and does not react with DPPH [102]. However, Fırat et al. (2018) reported the DPPH free radical scavenging effect of chamazulene isolated from chamomile volatile oil, with chamazulene being less active than ascorbic acid [103]. The ABTS scavenging effect of *A. millefolium* L. essential oil demonstrated in our study is supported by the observations of Capuzzo et al. (2014), who reported that chamazulene possesses an ABTS radical scavenging effect [102].

Yildirim et al. (2023) demonstrated that *A. millefolium* L. volatile oil has ABTS radical scavenging activity and supported the idea that camphor and borneol from the volatile oil are important for its antioxidant effect [24]. The results of our study are in accordance with those obtained by Mohammed et al. (2023), who reported that the reducing power of *A. millefolium* L. volatile oil, demonstrated by FRAP assay, is stronger than its ability to scavenge DPPH free radicals [57]. Čulum et al. (2022) reported a low antioxidant activity for the essential oil extracted from *A. millefolium* L. harvested from Bosnia and Herzegovina [30]. However, our results are aligned with their observation that this volatile oil is more effective in scavenge ABTS radicals than DPPH radicals. Efremov and Zykova (2022) reported a lower percentage of DPPH inhibition (52.8%) for the whole volatile oil of *A. millefolium* L. [104].

Our observation that *M. longifolia* L. volatile oil is a weaker DPPH and ABTS inhibitor than *T. serpyllum* L. volatile oil is aligned with the results of Torres-Martínez et al. (2018) [105]. They reported that pulegone and menthone (the two main constituents of *M. longifolia* L. essential oil investigated in the present study) are less effective as DPPH and ABTS free radical scavengers than thymol (the most abundant compound from the *T. serpyllum* L. essential oil tested in our study). Sharopov et al. (2014) also demonstrated that menthone had a lower DPPH scavenging effect than the thymol, carvacrol, and γ-terpinene constituents of *T. serpyllum* L. essential oil [106]. In the ABTS assay, carvacrol, p-cymene, linalool, γ-terpinene, and thymol performed better than menthone. Stronger DPPH inhibition of *M. longifolia* L. [106] volatile oil as compared with *A. millefolium* L. volatile oil can be attributed to the menthone content of *M. longifolia* L. Menthone demonstrated a higher DPPH scavenging effect than camphor and β-pinene, two constituents of *A. millefolium* L. volatile oil. In an ABTS test, 1,8-cineole and β-pinene, also present in the *A. millefolium* L. volatile oil investigated in our study, were more effective than menthone.

Nikšić et al. (2012) reported that *M. longifolia* L. volatile oil, with piperitone oxide being the most abundant compound, dose-dependently inhibited DPPH free radicals by 45.22–98.50% [107]. Abdel-Gwad et al. (2022) also reported that *M. longifolia* L. volatile oil inhibited the DPPH free radical, in a dose-dependent manner, by 35.52–65.82% [108]. Amar et al. (2025) proved the antioxidant activity of *M. longifolia* L. volatile oil by both DPPH and FRAP assay [109]. Our results differ from those reported by Tourabi et al. (2023), which observed that *M. longifolia* L. essential oil is more effective in scavenging ABTS free radicals than DPPH free radicals [36]. Since the oil analyzed by Tourabi et al. (2023) contained higher amounts of piperitenone oxide, caryophyllene, and (−) germacrene D, the difference in the radical scavenging activity can be explained by the variations in the chemical composition of the volatile oils [36].

To sum up, the in vitro antioxidant effect of the analyzed volatile oils varied by their chemical composition. The antioxidant effect is mainly attributed to the major volatile constituents, but the minor compounds can also induce synergistic or antagonistic effects. Essential oils also revealed various antioxidant capacities among different applied in vitro tests.

Compared to the in vivo tests for evaluating antioxidant potential, the in vitro evaluations present a number of limitations that are responsible for the differences observed between the results from these two types of assays. One reason for the observed differences is that the in vitro tests are based on simple, isolated chemical reactions which do not allow for replication of the physiological conditions of a living organism. Furthermore, the antioxidant effect is dependent on bioavailability as well as the metabolic transformations the substance undergoes before reaching its site of action. Nevertheless, in vitro methods enable a preliminary assessment of free radical scavenging potential or reducing power, serving as rapid and cost-effective techniques [110].

### 2.4. Antibacterial Activity

As the literature shows, there are many factors affecting the antibacterial action of the EOs, with geographical location as a very important factor [111,112]. This observation justifies the analysis of the EOs obtained from the plants presented in this research and our attempt to compare the results with those obtained by using EOs from different regions in Romania or elsewhere, as in the previous chapters.

**Figure 1:**
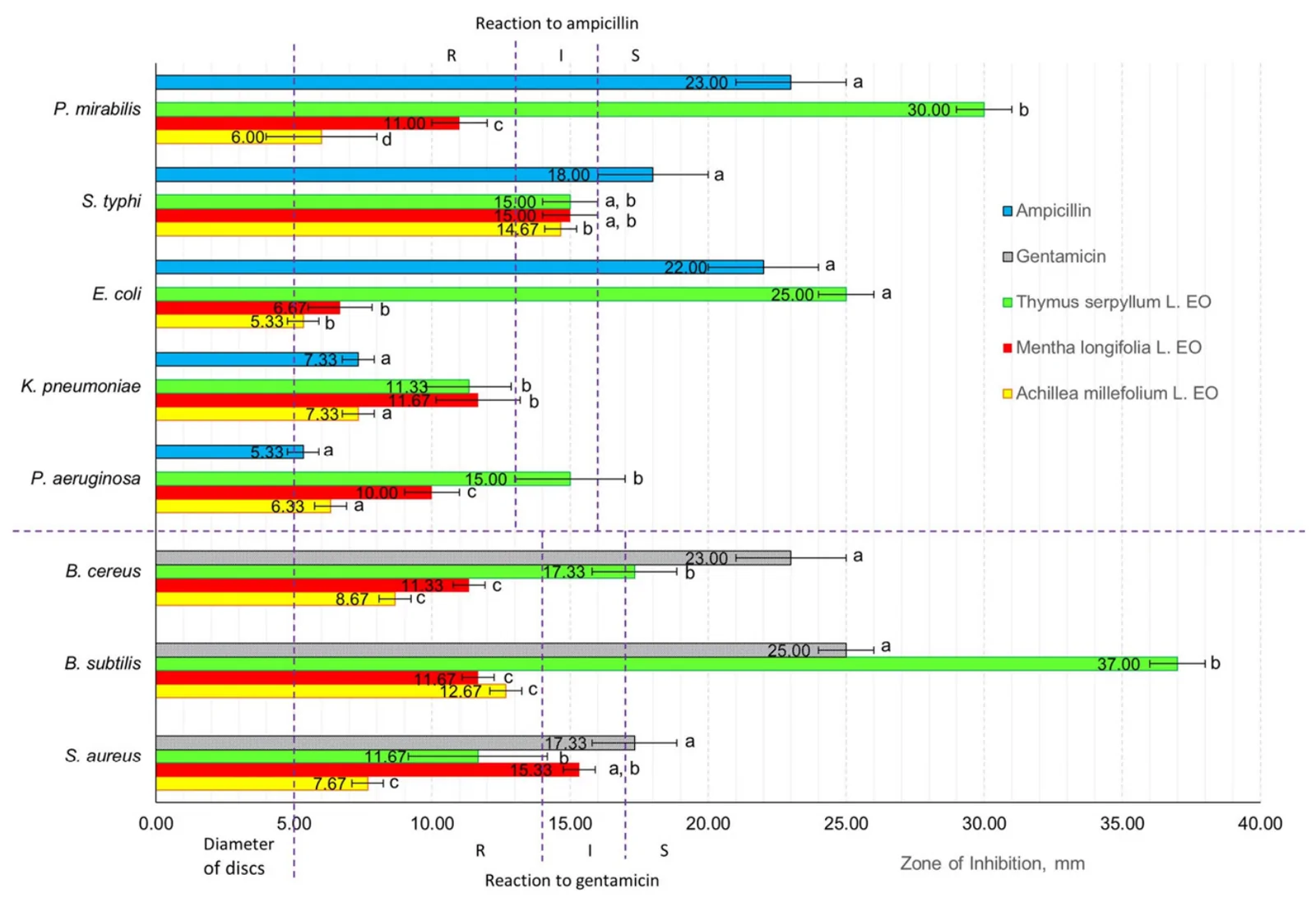
Antibacterial activity of the essential oils in terms of eight pathogenic bacteria and antibiotics. The values in the graphic are the median values obtained from three experiments in parallel. For the same microorganism, different letters after the bars indicate a significant difference between the mean values (*p* < 0.05). R: resistant; I: intermediate; S: susceptible.

Figure 1 presents the results obtained from analysis of the antibacterial action of the essential oils on the pathogenic bacteria selected for this investigation. The type of EO used affects the size of the inhibition zone. *Thymus serpyllum* L. has the most potent essential oil, featuring exceptional activity against *B. subtilis*, *E. coli*, and *P. mirabilis*, outperforming ampicillin and gentamicin, the standard antibiotics used here. Its powerful antibacterial properties stem primarily from its high concentrations of phenolic compounds, notably thymol and carvacrol [113], which were identified in high amounts in the EO obtained in this research. The EO from *Thymus serpyllum* L. has proven in vitro efficacy against both Gram-positive (e.g., *B. subtilis*) and Gram-negative (e.g., *E. coli*, *P. aeruginosa*, *S. enteritidis*) strains [113,114]. Only *S. aureus* and *K. pneumoniae* seem to be resistant to the EO from *Thymus serpyllum* L, with this result being in accordance with a study undertaken by Galovičová et al. (2021) [113] for *S. aureus*. This result is not consistent with other studies on commercial EO from *Thymus* species found on the Romanian market, showing the importance of different factors, with the two most important being the quantity of the EO used in the research found in the literature (10 μL in most of the studies, double the amount we used) [115,116] and the plant species from which the thyme oil was obtained (the EO from *Thymus vulgaris* behaves differently from *Thymus serpyllum* [117]); most of the Romanian-origin EOs investigated were commercial, having no clear provenance.

Previous studies demonstrated the synergistic effects of thymol and carvacrol mixtures against *E. coli* [118], and of carvacrol and cymene against *B. cereus* [119]. In another study, a mixture of carvacrol and thymol exhibited an additive effect against *E. coli* and *B. cereus* [120]. The findings support the sensitivity of these pathogens to the action of *T. serpyllum* L. essential oil. One of the antibacterial mechanisms of thymol and carvacrol is the disintegration of the bacterial cell membrane (the lipophilicity of the two compounds enables them to interact with the bacterial cell membrane, disrupting it and leading to the release of compounds from the cell). Other possible mechanisms include biofilm reduction, motility inhibition, inhibition of membrane adenosine triphosphatases, and inhibition of efflux pumps [121].

The EO from *Mentha longifolia* L. displays resistance across most strains, with intermediate action against both *S. aureus* and *S. typhi*; for *S. aureus*, the essential oil acts as a bactericide by altering membrane permeability, disrupting cell wall integrity, and interfering with amino acid metabolism [122]. There are studies which are not in accordance with our results, such as that of [36], which indicates this EO as efficient against *P. aeruginosa*, or that of [123], indicating it as efficient against *E. coli*, *S. aureus* and *B. subtilis*. Regarding the antibacterial action of the EO obtained from Romanian *Mentha longifolia* L. plants, studies from the literature seem to show similar results and indicate this plant as producing an EO resistant to *S. aureus* and *E. coli* [124]. Similar observations with respect to the case of the EO produced from *Thymus serpyllum* L. can be made, in terms of the importance of the intensity of the antibacterial action of the EO from *Mentha longifolia* L. arising from the quantity of the EO used in the analysis and origin of the plant [36,125] or, in this case, the extraction method employed [81].

The EO from *Achillea millefolium* L. shows resistance to most of the studied bacteria, with intermediate action recorded against *S. typhi* (Figure 1). Other similar studies showed low to intermediate antibacterial action with respect to this EO, similar to our analyses [24], even when the essential oil was used in a higher concentration (10 μL) [126]. As other research shows, the EO from Romanian *Achillea millefolium* L. plants seems to be not very efficient against bacteria [127].

As Figure 1 shows, gentamicin is highly effective against all the tested Gram-positive bacteria (*S. aureus*, *B. subtilis*, *B. cereus*), and ampicillin is highly effective against *E. coli* and *P. mirabilis* but shows little to no activity against *P. aeruginosa* and *K. pneumoniae*. An explanation as to why ampicillin is not efficient against *P. aeruginosa* is given by its low outer membrane permeability, the presence of active efflux pumps, and the production of specific antibiotic-inactivating enzymes like AmpC β-lactamase [128]. No efficiency of ampicillin is obtained against *K. pneumonia*, because the bacteria naturally produces a constitutive chromosomal β-lactamase enzyme known as SHV-1, which breaks down the antibiotic [129].

### 2.5. The Effect of Volatile Oils on the Viability of Drosophila Melanogaster

Considering that assessing the toxicity of essential oils is a crucial step required before introducing such natural products in therapy, we conducted an in vivo experiment to investigate the viability of fruit flies fed with a normal diet supplemented with *A. millefolium* L., *M. longifolia* L., and *T. serpyllum* L. essential oils at different concentrations. All *Drosophila melanogaster* individuals used for this test were the same age and had identical genotypes and epigenomes. Since studies on human subjects are strongly influenced by genetic and age variability, our study could be a starting point for the design of clinical trials [13].

**Figure 2:**
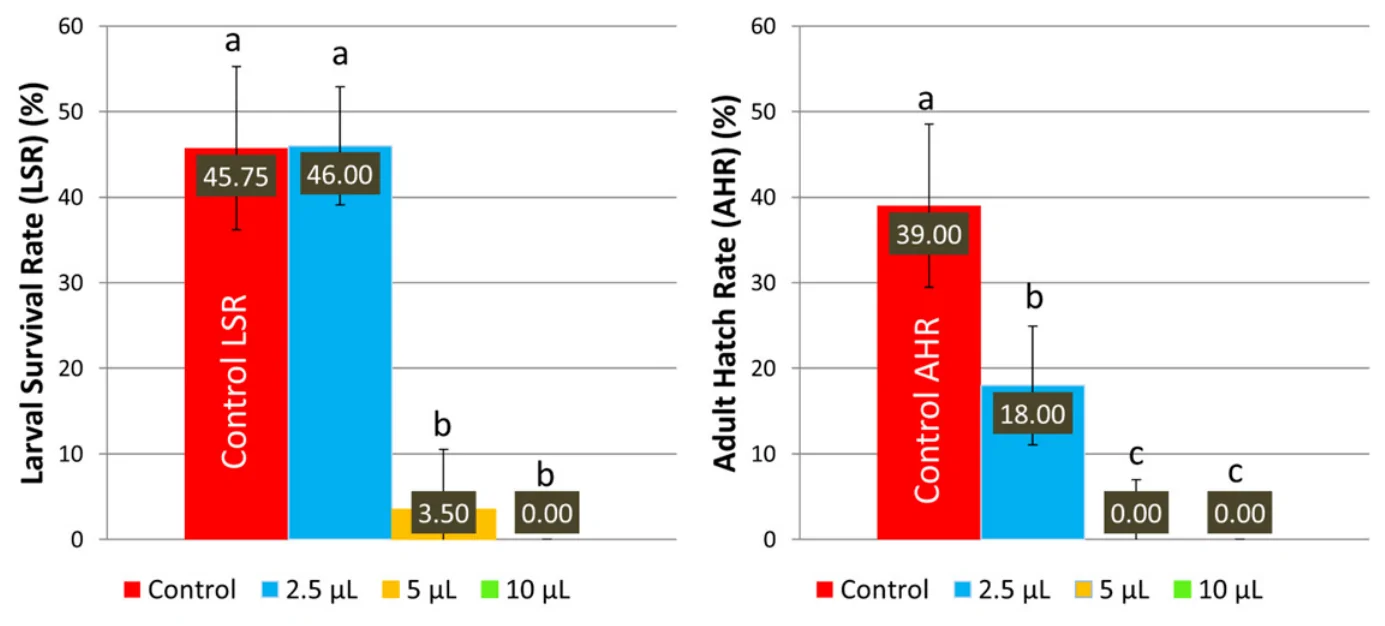
Larval survival and adult hatching rates in normal medium supplemented with *A. millefolium* L. volatile oil. Different letters indicate a significant difference between means (*p* < 0.05).

Our research demonstrated that *Drosophila melanogaster* reared on a normal diet supplemented with 2.5 μL *A. millefolium* L. volatile oil per 4 mL of medium showed no significant difference regarding larval survival rate compared to the control group (Figure 2). A significant decrease in larval survival rate was observed in the groups treated with *A. millefolium* L. volatile oil at 5 and 10 μL per 4 mL of normal medium, the latter being completely toxic. A significant decrease in the percentage of hatched adult flies in the group treated with 2.5 μL per 4 mL of normal medium was observed compared to the control, which suggested pupae mortality even at the lowest tested concentration. No hatched adult flies were observed in the groups treated with 5 and 10 μL of *A. millefolium* L. volatile oil per 4 mL of normal medium.

**Figure 3:**
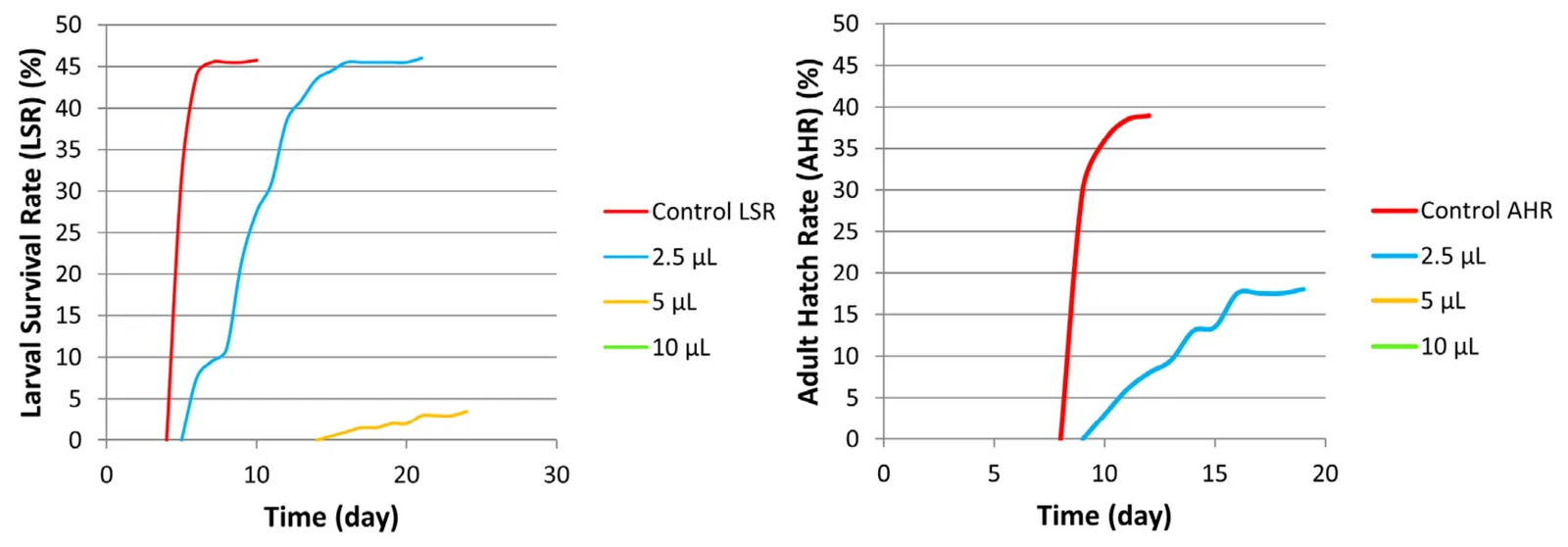
Developmental time of *Drosophila melanogaster* in normal medium supplemented with *Achillea millefolium* L. volatile oil.

Along with viability, developmental time of fruit flies was studied. A one-day delay in the development of larvae to pupae and pupae to adult stages, respectively, was observed in the group treated with 2.5 μL of *A. millefolium* L. volatile oil per 4 mL of normal medium compared to the control group. In this case, the larval survival rate was not affected, but a significant decrease in adult hatch rate compared to the control was observed (Figure 3). Furthermore, at a concentration of 5 μL of volatile oil per 4 mL of normal medium, a ten-day delay was found compared to the control, with the viability decreasing from 45.75% in the control group to 3.50% in the tested group.

Regarding chamazulene, the main constituent of *A. millefolium* L. volatile oil analyzed in the present research, to our knowledge, no studies have been conducted to demonstrate the toxic effect of this compound against *Drosophila melanogaster*. However, Zhang et al. (2016) demonstrated the insecticidal effect of monoterpenes and their oxygenated derivatives [130]. They reported that some of the volatile constituents identified in *A. millefolium* L. volatile oil investigated in our study, such as (–)-α-pinene, (–)-β-pinene, myrcene, γ-terpinene, α-terpineol, (±)-terpinen-4-ol, and 1,8-cineol, had toxic effects against *Drosophila melanogaster*. Therefore, these toxic effects could be attributed to the volatile oil composition.

**Figure 4:**
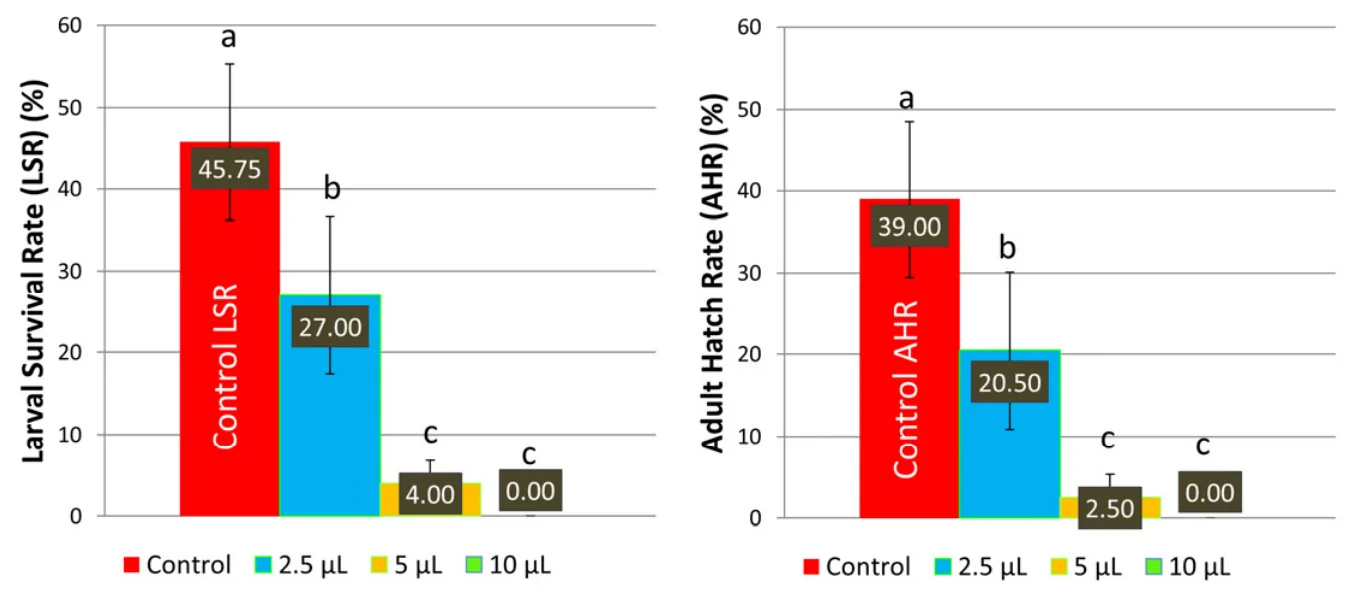
Larval survival and adult hatching rates in normal medium supplemented with *Mentha longifolia* L. volatile oil. Different letters indicate a significant difference between means (*p* < 0.05).

In the case of *M. longifolia* L. volatile oil, a significant decrease in the viability of larvae and adult flies compared to the control was observed at all tested concentrations, with the highest one causing total lethality (Figure 4).

**Figure 5:**
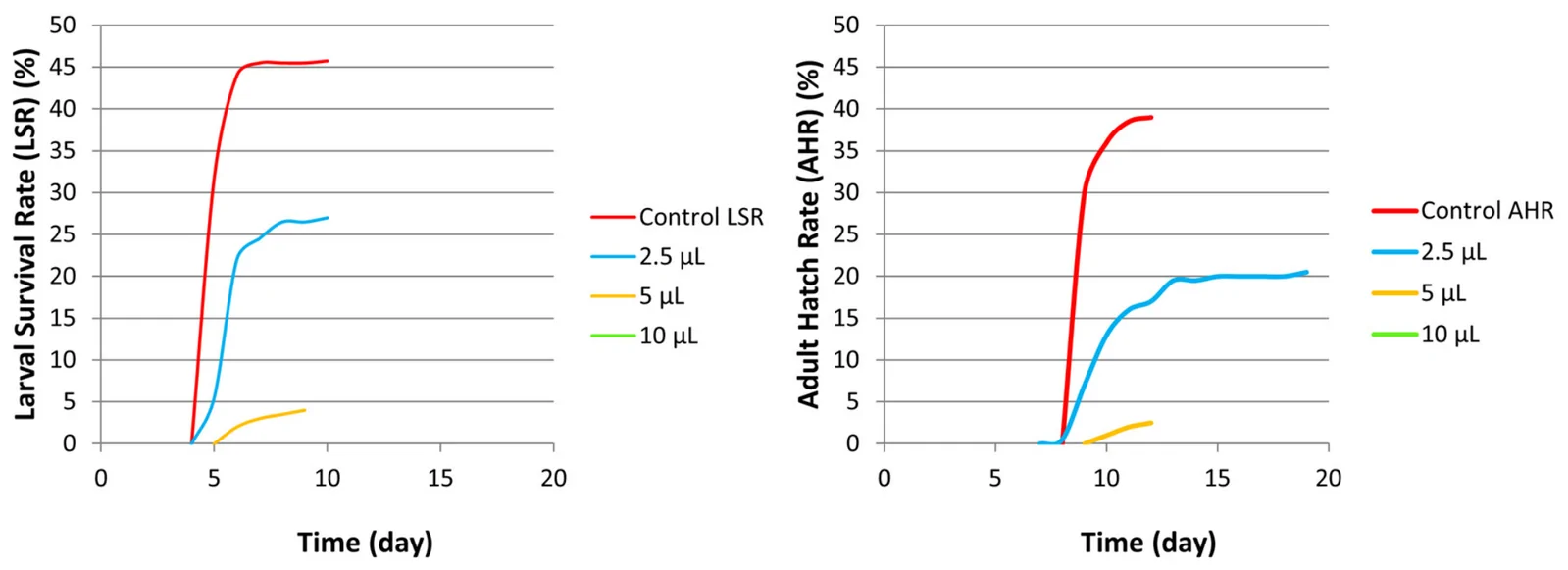
Developmental time of *Drosophila melanogaster* in normal medium supplemented with *Mentha longifolia* L. volatile oil.

Regarding developmental time, a normal diet supplemented with 5 μL of *M. longifolia* L. volatile oil per 4 mL of medium caused a one-day delay in larvae to pupae development, whereas 2.5 μL of *M. longifolia* L. volatile oil per 4 mL of medium did not change the developmental time (Figure 5). Since viability was null at a concentration of 10 μL of *M. longifolia* L. volatile oil per 4 mL of nutritive medium, monitoring developmental time was impossible in this case.

A previous study conducted by Zhang et al. (2016) found that (+)-pulegone and (–)-menthone, some of the major constituents of the *M. longifolia* L. volatile oil analyzed in the present study, exerted toxic effects against *Drosophila melanogaster*, pulegone being the second most toxic volatile oil component investigated in that study, after (±)-citronellal [130].

**Figure 6:**
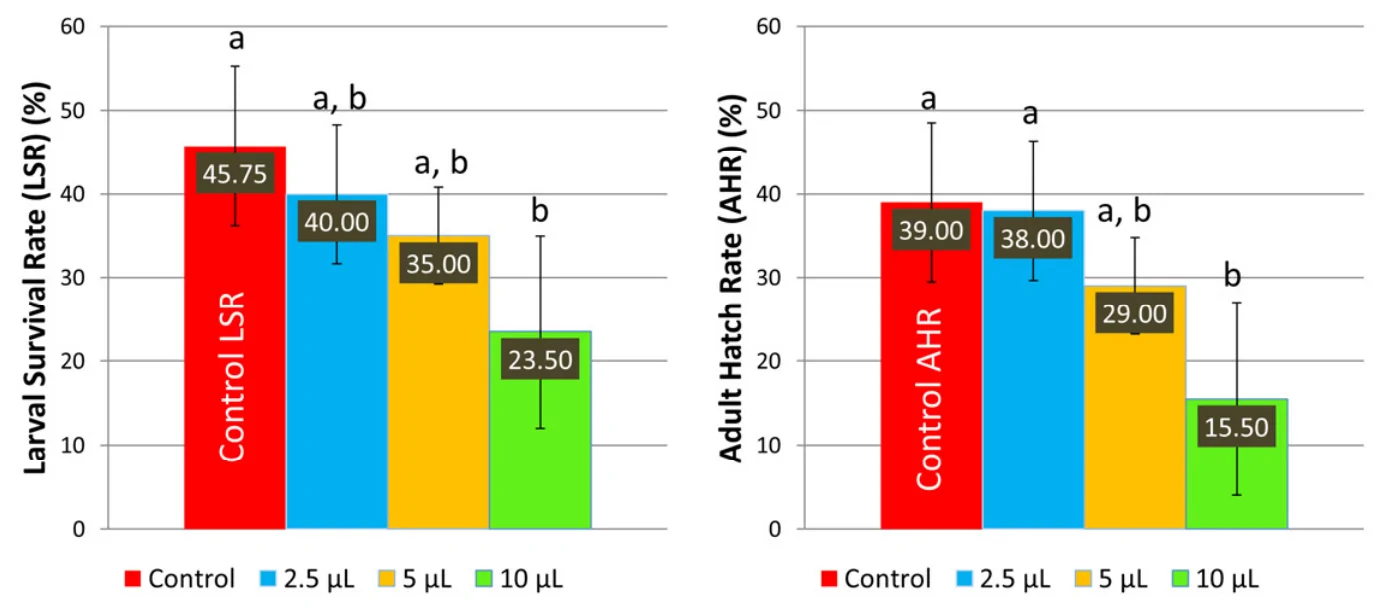
Larval survival and adult hatching rates in normal medium supplemented with *Thymus serpyllum* L. volatile oil. Different letters indicate a significant difference between means (*p* < 0.05).

The viability of *Drosophila menanogaster* treated with *T. serpyllum* L. volatile oil at concentrations of 2.5 and 5 μL per 4 mL of normal medium showed no significant differences compared to the viability of the control group (Figure 6). At a concentration of 10 μL of *T. serpyllum* L. volatile oil per 4 mL of normal medium both larval survival and adult hatch rates were significantly decreased.

**Figure 7:**
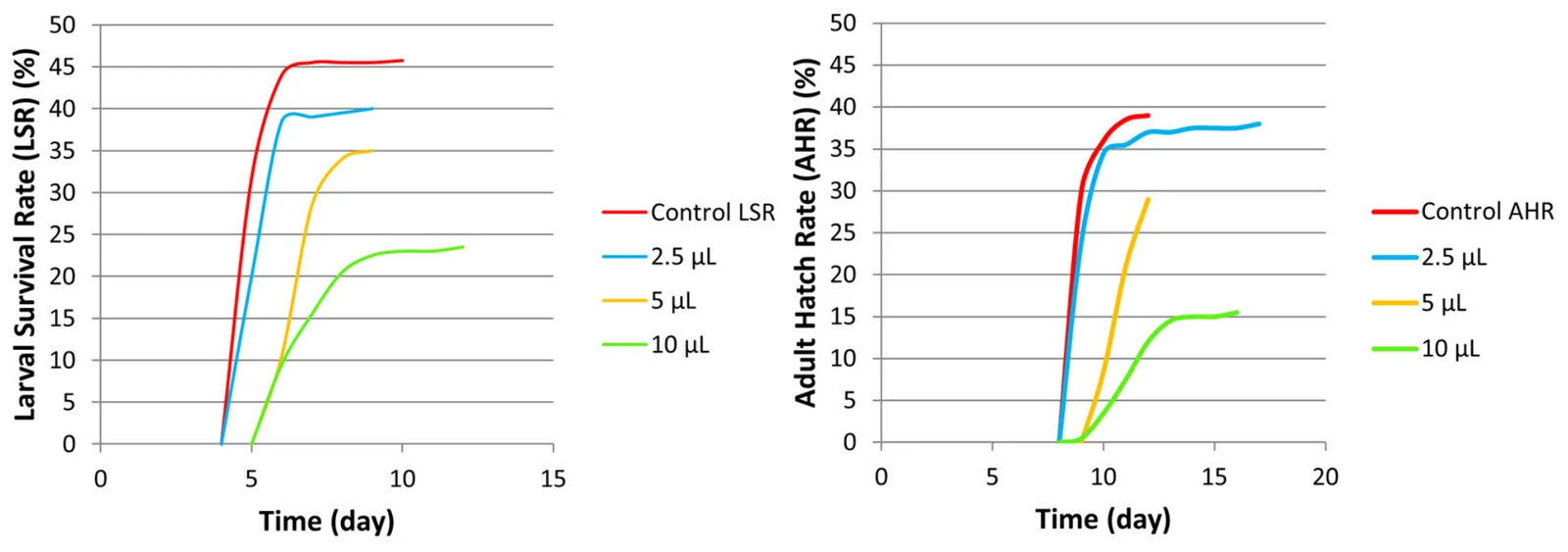
Developmental time of *Drosophila melanogaster* in normal medium supplemented with *Thymus serpyllum* L. volatile oil.

In the group of *Drosophila melanogaster* fed with normal diet supplemented with 2.5 μL of *T. serpyllum* L. volatile oil per 4 mL of normal medium, no delay in the development of larvae and adult flies was observed (Figure 7). A one-day delay was recorded in the development of larvae and adult flies at a concentration of 5 and 10 μL per 4 mL of normal medium, with the exception of the group of adult flies fed with 10 μL of *T. serpyllum* L. volatile oil per 4 mL of normal media, for which no difference in developmental time was observed.

Since thymol was the most abundant volatile compound in the *T. serpyllum* essential oil analyzed in our study, it could be responsible for the toxic effect against *Drosophila melanogaster*, because previous research conducted by Erdem Kucuk and Kaplan (2023) demonstrated that thymol in concentrations ranging from 10 to 1000 mg/L negatively impacts the survival rates of larvae, pupae and adult fruit flies, while extending the developmental time [131]. The authors associated the decreased survival rate of adult individuals with reduced nutrient consumption after exposure to a concentration of 1000 mg thymol/L. Sousa Silveira et al. (2020) also found that thymol and carvacrol, another major constituent of *T. serpyllum* L. volatile oil investigated in the present study, had significant toxic effect against *Drosophila melanogaster* and significantly affected the locomotor function of fruit flies at concentrations of 31 μg thymol/mL and 30 μg carvacrol/mL [132]. Similar results were reported by Zhang et al. (2016), who demonstrated that oxygenated monoterpenes, such as thymol and carvacrol, exerted a strong toxic effect against *Drosophila melanogaster* [130]. They also observed that monoterpene hydrocarbons, such as p-cymene and γ-terpinene, exert toxic effects against fruit flies [130]. Based on these findings, the toxic effect of *T. serpyllum* L. volatile oil could be attributed to its major constituents, especially thymol and carvacrol, but other major and minor constituents could also be involved.

The biological effects of volatile oils are often attributed to their major compounds. However, given that essential oils are complex mixtures of various substances, it must be highlighted that the effect may result from interactions between them—whether synergistic, additive, or antagonistic [133]. Although numerous studies in the literature have analyzed the toxicity of volatile oils against *D. melanogaster*, studies evaluating the potential interactions of volatile oil components are limited. However, there are studies that evaluated interactions between volatile oil components in other insects [134,135,136]. A previous study demonstrated that there are no significant differences between the toxicity of *Salvia fructicosa* essential oil and mixtures of individual compounds (such as 1,8-cineol in combination with tujone and 1,8-cineol in combination with camphor) in concentrations relative to those in the volatile oil, both against *D. melanogaster* and *B. oleae* [134]. On the other hand, many synergistic and additive interactions were observed between volatile oils individual compounds when tested against *Musca domestica* and *Spodoptera littoralis* [135,136]. Antagonistic interactions were also reported, such as in the case of mixtures of pulegone and menthone [134], eugenol and thymol [135], and carvacrol and 1,8-cineol [136]. It is worth noting that minor compounds also play a significant role in the emergence of toxic effects, as observed with *Salvia fructicosa* and *Mentha pulegium* volatile oils, which demonstrated stronger toxicity than binary mixtures of their major compounds [134].

**Figure 8:**
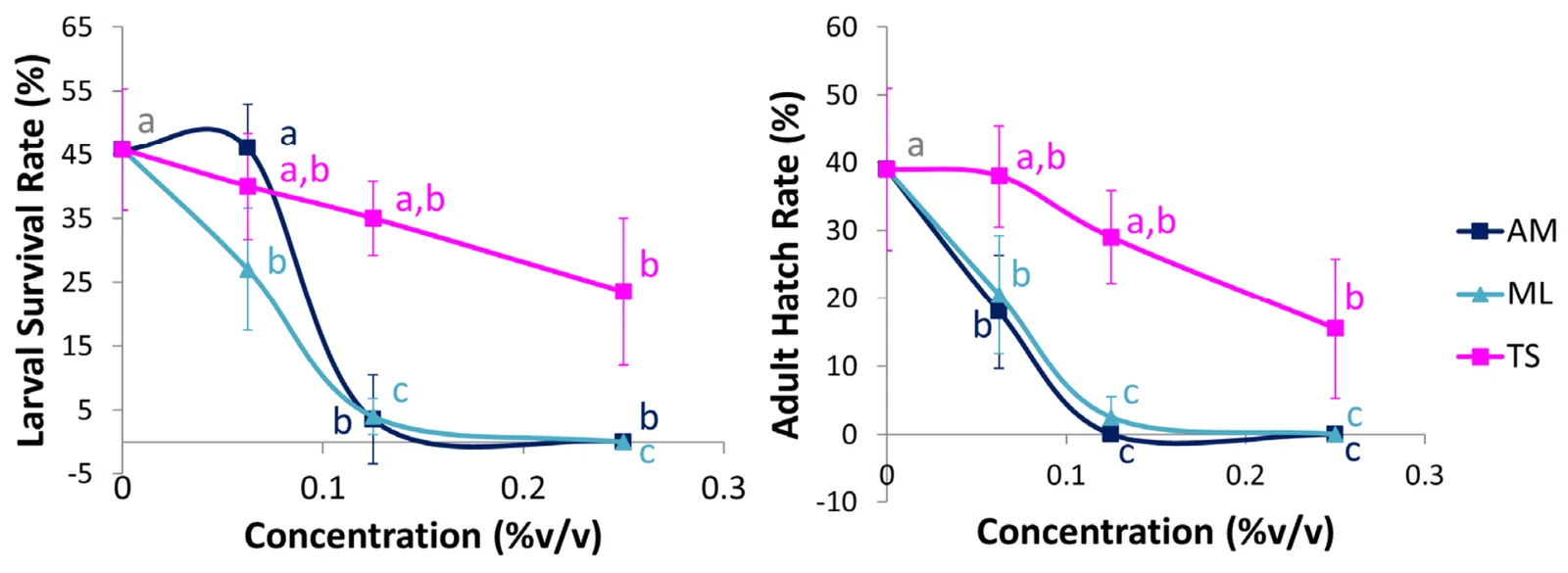
Comparative larval survival and adult hatching rates in normal medium supplemented with *Achillea millefolium* L. (AM), *Mentha longifolia* L. (ML), and *Thymus serpyllum* L. (TS) volatile oils. Data are presented as mean ± SD (*n* = 4). Means representing the same volatile oil followed by different letters are significantly different (*p* < 0.05).

Considering the above mentioned results, all tested volatile oils in concentrations ranging from 0.0625 to 0.25% demonstrated a dose-dependent decrease in the viability of *Drosophila melanogaster* in different developmental stages (Figure 8). *A. millefolium* L. volatile oil showed the most toxic effect against *Drosophila melanogaster*, followed by *M. longifolia* L. and *T. serpyllum* L. essential oils. The highest tested concentration of 0.25% has a significantly toxic effect, inducing total mortality of larvae and pupae in the groups fed with *A. millefolium* L. and *M. longifolia* L. volatile oils, respectively. *T. serpyllum* L. volatile oil at a concentration of 0.25% significantly decreased the larval survival rate to 23.5% and adult hatch rate to 15.5%. At a concentration of 0.125%, *A. millefolium* L. essential oil was the most toxic among the tested volatile oils, revealing a significant lower viability compared to the control. Similar toxicity, but with slightly higher survival rates, was observed for *M. longifolia* L. essential oil. In contrast to the first essential oils, the volatile oil of *T. serpyllum* L. at 0.125% did not significantly influence larval survival and adult hatch rates compared to the control. Interestingly, at a concentration of 0.0625%, *A. millefolium* L. volatile oil showed the highest larval survival rate, followed by *T. serpyllum* L. and *M. longifolia* L. volatile oils, the first two showing no difference compared to the control group. Adult hatch rates at 0.0625% volatile oil increased in the following order: *A. millefolium* L., *M. longifolia* L., and *T. serpyllum* L., with no significant difference being observed between the latter and the control group.

Compared to the results of our previous study, in which we assessed the viability and developmental time of *D. melanogaster* fed with normal media supplemented with different concentrations of *A. millefolium* L., *M. longifolia* L. and *T. serpyllum* L. aerial part extracts [137], we observed that for the volatile oils, the toxic effect occurred at lower concentrations. This fact could be explained in relation to the compounds which were quantified in the volatile oils and previously reported to exert toxic effects against fruit flies, such as (–)-α-pinene, (–)-β-pinene, myrcene, γ-terpinene, α-terpineol, (±)-terpinen-4-ol, 1,8-cineole, (+)-pulegone, (–)-menthone [130], thymol [130,131,132], and carvacrol [130,132].

To the best of our knowledge, this is the first study investigating the toxicity of *A. millefolium* L., *M. longifolia* L., and *T. serpyllum* L. essential oils against *Drosophila melanogaster*, making it impossible to compare our results with those obtained by other researchers. However, our results are in accordance with those reported by Bernardes et al. (2023), who studied the toxicity of *Lavandula angustifolia*, *Zingiber officinale*, and *Copaifera* sp. against *Drosophila melanogaster* [15]. They showed a dose-dependent decrease in survival rates in *Drosophila melanogaster* fed with nutritive medium supplemented with volatile oil concentrations ranging from 0.0625 to 0.5% (%*v*/*v*). Lifespan was shorter in the group treated with 0.0625% *Zingiber officinale* essential oil compared to the control, whereas 0.125% *Lavandula angustifolia* volatile oil extended lifespan by 12 days. *Copaifera* sp. at 0.0625% did not influence the lifespan of *Drosophila melanogaster*. Bouabida and Dris (2022) also observed a dose-dependent increase in the mortality of *Drosophila melanogaster* third instar larvae after 24, 42, and 72 h of exposure to *Ruta graveolens* volatile oil [138]. Our results align with the observations of Mitić et al. (2022), who demonstrated that exposure of *Drosophila melanogaster* to *Abies* sp. volatile oils at concentrations ranging from 0.19 to 3% resulted in prolonged development time and reduced pupation and adult individual emergence, mainly at a concentration of 3% [14]. As we observed high pupae mortality in the case of *A. millefolium* volatile oil, they reported that *Abies cephalonica* volatile oil at a concentration of 3% led to the highest pupae mortality, with a lower percentage of adult hatch rates than the percentage of pupation. Similar to our results, Aksakal et al. (2012) showed that the larval survival rate of *Drosophila melanogaster* decreased in a dose-dependent manner when treated with *Cymbocarpum erythraeum* volatile oil [139].

Many studies in the specialized literature have demonstrated the toxic effects of essential oils on *D. melanogaster* without investigating the underlying mechanisms [14,15,138,139]. Consistent with the results observed in the present study, *R. officinalis* L. essential oil increased mortality among *D. melanogaster* larvae and adults while also impairing their development. Evaluation of oxidative stress biomarkers revealed that exposing fruit flies to *R. officinalis* L. essential oil induced a redox imbalance, increased lipid peroxidation, reduced thiol levels and superoxide dismutase activity, and elevated catalase and glutathione S-transferase activities. Given that certain predominant compounds found in *R. officinalis* L. volatile oil (α-pinene, β-pinene, 1,8-cineole, camphor, and camphene) are also present in the essential oils analyzed in these studies, it is possible that the toxic effect is mediated by this mechanism [140].

**Figure 9:**
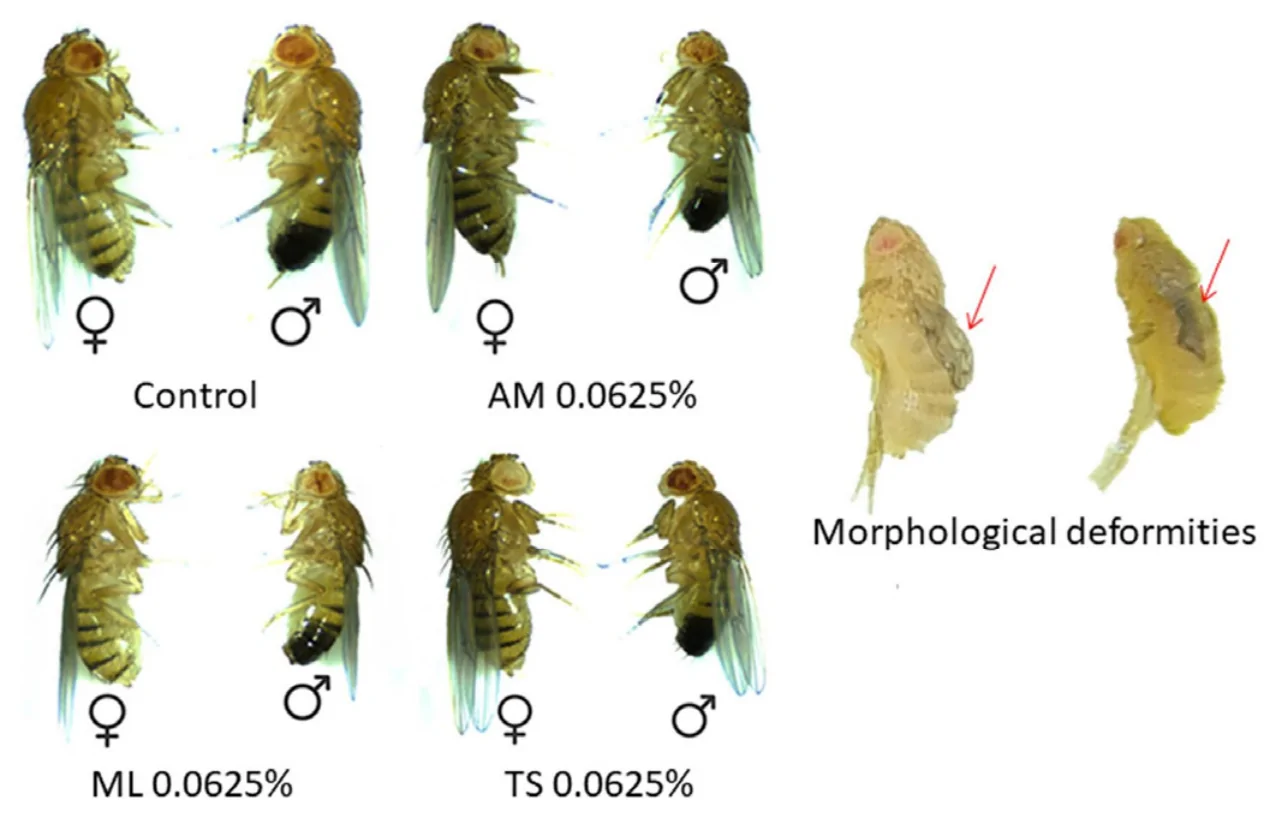
Volatile oil toxicity-induced morphological deformities in *Drosophila melanogaster*. AM: *Achillea millefolium* L. volatile oil; ML: *Mentha longifolia* L. volatile oil; TS: *Thymus serpyllum* L. volatile oil.

As compared to the control and the smallest concentration, in the group treated with higher concentrations (0.125 and 0.25%), morphological abnormalities were observed, such as incomplete development of body, legs, and head, and smaller and wrinkled wings, which led to the inability to fly (Figure 9).

Similar to our observations, Bouabida and Dris (2022) also reported physical abnormalities such as smaller pupae, incomplete pupation, and wrinkled wings in *Drosophila melanogaster* fed with *Ruta graveolens* volatile oil [138]. In a previous study, Khater (2021) reported deformities in *Lucilia cuprina* larvae, pupae, and adult flies emerging after exposure to *Mentha longifolia* volatile oil at concentrations ranging from 0.5 to 7% [141].

The results show that the tested volatile oils exerted both beneficial and toxic effects. This study is a first step in a wider research plan which involves the development of a pharmaceutical product containing plant extracts and volatile oils. At this stage, the safety of the volatile oils is not guaranteed. Also, future investigations on other model organisms and clinical studies are needed to establish efficient and safe doses for humans.

## 3. Materials and Methods

### 3.1. Chemicals and Reagents

Anhydrous sodium sulfate, hexane and high purity He were obtained from Merck Romania SRL, Bucharest, Romania. Methanol, acetic acid, DPPH, and 2,4,6-tripyridyl-s-triazine (TPTZ) were purchased from Sigma-Aldrich Company, St. Louis, USA. Potassium persulfate, charcoal, agar powder, and saccharose were acquired from VWR Chemicals, Leuven, Belgium. Sodium acetate, ferric chloride, hydrochloric acid, and ascorbic acid were purchased from Chemical Company, Iași, Romania. Trolox, ABTS, and nipagin were obtained from Thermo Fisher Scientific, Kandel, Germany.

Mueller–Hinton for producing the bacterial inocula and Mueller–Hinton agar for the antimicrobial susceptibility tests (ASTs) were purchased from Merck Romania SRL, Bucharest, Romania. ROTI^®^Antibiotic Discs Gentamycin (10 µg) for microbiology and Oxoid™ Ampicillin Antimicrobial Susceptibility discs (10 µg) were purchased from Carl Roth GMBH, Karlsruhe-Mühlburg, Germany, and Thermo Fisher Scientific, Kandel, Germany, respectively.

### 3.2. Plant Collection and Pre-Extraction Treatment

Aerial parts of aromatic plants included in this study were collected from unpolluted mountainous areas of Sibiu County, Romania (*Achillea millefolium* L.—45°41′20″ N, 23°59′9″ E, 1130 m; *Mentha longifolia* L.—45°41′22″ N, 23°59′29″ E, 1100 m; and *Thymus serpyllum* L.—45°42′34″ N, 24°1′33″ E, 630 m), in July 2021. Identification of plant species was performed according to “Vascular Plants of Romania, illustrated field guide” by Sârbu et al. (2013) [142]. A voucher specimen for each plant (no. 101/2—*A. millefolium* L., no. 101/3—*Mentha longifolia* L., no. 101/4—*Thymus serpyllum* L.) was deposited at the “Lucian Blaga” University of Sibiu, Romania, in the Faculty of Medicine, pharmacy specialization. The collected plant products were shade-dried at room temperature, preserved and protected from light in labeled paper bags until the time of extraction. Shredded aerial parts were kept in warm water for 12 h before extraction to promote wetting of cells and to facilitate the release of oils during extraction.

### 3.3. Volatile Oils Extraction

Volatile oils were extracted from plant aerial parts by hydrodistillation in a neo-Clevenger apparatus modified by Moritz. Each extraction took about 5 h. The method of extraction was adapted according to the method described in the Romanian Pharmacopoeia, Xth Edition [143]. All extractions were performed in triplicate. After extraction, the volatile oils were kept in sealed brown glass vials, in the presence of anhydrous sodium sulfate, to separate them from water. Then, they were refrigerated at 4 °C until analysis. The percentage of volatile oil content (*VOC*%) in each sample was calculated according to Formula (1):

(1) $$VOC\%=\frac{obtained\;volatile\;oil\;(\mathrm{mL})}{dried\;plant\;used\;(g)}\times 100$$

### 3.4. GC-MS Profiling of Volatile Components

The method for analysis of the chemical percentage composition of volatile components was adapted after Păun et al. (2023) [144]. We used a gas chromatograph equipped with a mass spectrometry system, Agilent type 7890A/5975/2008, manufactured by Agilent Technologies, Inc. Europe, Waldbronn, Germany. A Phenomenex capillary column, type HP5-MS, with the following characteristics was used: length—30 m; interior diameter—0.25 mm; and thickness of stationary phase—0.25 μm. The sample was obtained by dissolving 10 μL of volatile oil in 1 mL of hexane. High purity He was used as a carrier gas. A volume of 1 μL of the sample was injected into the inlet of the chromatograph, set on „scan” mode, at a constant temperature of 250 °C. The carrier-gas flow rate was 1 mL/min. For the analysis, the temperature was kept at 40 °C during the first minute. After that, the temperature increased by 5 °C/min until it reached 220 °C. Then, the temperature increased by 20 °C/min until it reached 280 °C. The temperature was held at 280 °C for 5 min. The NIST L14 library was used to identify the compounds based on their retention time. All tests were performed in triplicate. The results were expressed as a percentage of each volatile oil component from the total of identified volatile compounds (%area).

### 3.5. Antioxidant Capacity

#### 3.5.1. DPPH Assay

A DPPH radical scavenging activity assay was performed by spectrophotometric method, using a Shimadzu 1900 UV-VIS spectrophotometer (Shimadzu, Kyoto, Japan). The method was adapted after Tylkowski et al. [145]. A 25 μg/mL DPPH standard solution was prepared, using methanol as a solvent. It was kept in a dark place for 2 h before the procedure.

A volume of 60 μL of undiluted oil was mixed with 1940 μL of 25 μg/mL DPPH solution. The absorbance of samples at 515 nm was recorded. A 1 mg/mL ascorbic acid solution was used as a positive control.

For the calibration curve, solutions with concentrations ranging from 0.5 to 25 μg DPPH/mL were prepared by diluting the stock solution with methanol and analyzed as samples. The calibration curve was plotted.

The final concentration of DPPH (*C_f_*), after it reacts with the sample, was calculated using Formula (2):

(2) $$C_{f}=\frac{A_{s}-i}{s}$$

where

*A_s_* is the absorbance of the sample;

*i* is the intercept of the calibration curve;

*s* is the slope of the calibration curve.

The results were expressed as a percentage of DPPH free radical scavenging activity (*RSA*), which was determined by Equation (3):

(3) $$RSA\;(\%)=\frac{C_{i}-C_{f}}{C_{i}}\times 100$$

where *C_i_* is the DPPH concentration of the standard solution and *C_f_* is the DPPH concentration of the solution after it reacts with the sample, both expressed as μg/mL.

All tests were performed in triplicate.

#### 3.5.2. ABTS Assay

An ABTS assay was performed by spectrophotometric method, using a Shimadzu 1900 UV-VIS spectrophotometer (Shimadzu, Kyoto, Japan). The method was adapted after Vicaș et al. (2015) [146]. A 7 mM ABTS and 2.45 mM potassium persulfate stock solution was prepared and kept protected from light, at room temperature, for 16 h before analysis. Then, the stock solution was diluted until its absorbance at 734 nm was 0.7 ± 0.02.

A volume of 25 μL of the sample was mixed with 2.5 mL diluted stock solution and vortexed for 30 s. After 1 min, its absorbance was measured at 734 nm. A 1 mg/mL ascorbic acid solution was used as a positive control.

For the calibration curve, solutions with concentrations ranging from 0.125–2 mmol Trolox/L were prepared and analyzed as samples. The calibration curve was plotted. The results were expressed as mmol Trolox equivalent (TE)/mL volatile oil. All tests were performed in triplicate.

#### 3.5.3. FRAP Assay

An FRAP assay was performed by spectrophotometric method, using a Shimadzu 1900 UV-VIS spectrophotometer (Shimadzu, Kyoto, Japan). The method was adapted after Vicaș et al. (2015) [146]. A working solution consisted of 50 mL of 300 mM acetate buffer with pH = 3.6, 5 mL of 20 mM FeCl_3_ solution, and 5 mL of 10 mM TPTZ solution acidified with 0.15 mL of HCl.

A volume of 100 μL of the sample was mixed with 500 μL of the working solution and 2 mL of purified water, then kept in a dark place for an hour. The absorbance at 595 nm was recorded. A 1 mg/mL ascorbic acid solution was used as a positive control.

For the calibration curve, solutions with concentrations ranging from 0.15–0.5 μmol Trolox/mL were prepared and analyzed as samples. The calibration curve was plotted. The results were expressed as μmol TE/mL volatile oil. All tests were performed in triplicate.

### 3.6. Antibacterial Activity

The selection of the pathogens was based on future possible applications of the EOs in food [147] or in medicine [148,149]; in this respect, six foodborne pathogens (*Staphylococcus aureus* ATCC 25923, *Bacillus cereus* ATCC 12600, *Escherichia coli* ATCC 11775, *Salmonella typhi* ATCC 1408, *Proteus mirabilis* ATCC 35659, and *Pseudomonas aeruginosa* ATCC 27853) and two opportunistic bacteria in hospitals *(Bacillus subtilis* ATCC 6051 and *Klebsiella pneumoniae* ATCC 43816) were chosen. Three of the bacteria were Gram-positive (*B. cereus*, *B. subtilis* and *S. aureus*) and the other five Gram-negative (*P. aeruginosa*, *K. pneumoniae*, *E. coli*, *S. typhi* and *P. mirabilis*).

The bacterial inocula were prepared as described in Georgescu et al. (2022) [150], by cultivating pure cultures of pathogens into Mueller–Hinton at 37 °C for 48 h, followed by the standardization of the suspensions to a 0.5 McFarland index.

ASTs were done to measure the antimicrobial EOs’ ability to inhibit the growth of selected microorganisms. The antimicrobial activity of the essential oils was analyzed by adapting the Kirby–Bauer disk diffusion method as presented in Georgescu et al. (2022) [150] with the following modifications: The quantity of undiluted EOs used in the analyses was 5 µL/ disc; a gentamicin 10 µg/disc and ampicillin 10 µg/disc were served as positive standard controls for Gram-positive and Gram-negative bacteria. The Zones of Inhibition (ZOIs) around the discs, in millimeters, were measured by using the AxioVision Rel 4.8 measuring software (Carl Zeiss Microscopy GMBH) and a stereo microscope with MicroCam 5.0 (Zeiss Stemi 508, Carl Zeiss Gottingen, Germany). ZOIs were interpreted according to the Clinical and Laboratory Standards Institute (CLSI) criteria, as follows:

- Ampicillin 10 μg/disc: Sensitive for ZOI ≥17 mm; intermediate for ZOI = 14–16 mm; resistant for ZOI ≤ 13 mm;
- Gentamicin 10 μg/disc: Sensitive for ZOI ≥18 mm; intermediate for ZOI = 15–17 mm; resistant for ZOI ≤ 14 mm [151].

### 3.7. In Vivo Drosophila Melanogaster Viability Assessment

The volatile oils were tested according to a method adapted after Aleya et al. (2023) [17]. To perform the experiment, a culture medium with normal saccharose content (normal medium) was prepared. An amount of 70 g of yeast paste was added to 1.2 L of water and mixed until homogeneous. Next, 52 g of saccharose and 30 g of wheat flour were added. The resulting mixture was brought to boil, under continuous stirring, after which 10 g of agar powder was added. It was boiled for 30 min under continuous stirring. After cooling to 50 °C, 1 g nipagin was added and mixed until homogeneous.

Three concentrations of volatile oil were tested (0.0625, 0.125 and 0.25, %*v*/*v*). A volume of 4 mL of culture medium was added to the tubes, over which different volumes of volatile oil (2.5, 5, and 10 μL, respectively) were pipetted. Contents of the tubes were mixed with a spatula for homogenization. Tubes were left overnight to solidify the medium. All experiments were performed in quadruplicate. Choosing of tested concentrations was based on previous studies reported in the literature which analyzed the toxicity of different volatile oils against *Drosophila melanogaster*. One of the studies conducted by Bernardes et al. (2023) tested the toxicity of three essential oils against *Drosophila melanogaster* at concentrations ranging from 0.0625 to 0.5% [15], while Mitić et al. (2022) tested the toxicity against *Drosophila melanogaster* of essential oils extracted from three coniferous species at concentrations ranging from 0.19 to 3% [14].

Obtaining *Drosophila melanogaster w^m4h^* embryos was accomplished by placing about 200 5-day-old female and male individuals into an egg collector fixed over a Petri dish with zero medium. This medium was prepared by mixing 1 g of charcoal with 1 g of agar powder and 100 mL of distilled water. Distilled water was initially boiled, and then charcoal and agar powder were successively added. The resulting mixture was boiled for 30 s and then cooled to 45 °C. In the center of the plate, a spatula tip of yeast paste was added to the surface of the medium. The Petri dish of the collector was changed every two hours. The whole process was carried out at 25 °C, in controlled humidity conditions [17].

After solidification of the medium, 50 *Drosophila melanogaster w^m4h^* 0–2-h-old embryos were added to each tube using fine tweezers. The new pupae and flies were counted daily [17].

### 3.8. Statistical Analysis

The results are expressed as mean and standard deviation (SD). Statistical differences between the means were evaluated by one-way ANOVA (Tukey’s post hoc test). All statistical tests were computed using IBM SPSS Statistics, version 26 (IBM Corp., Armonk, NY, USA).

## 4. Conclusions

This research highlights the chemical composition, antimicrobial and antioxidant activities, and toxicity against *Drosophila melanogaster* of three essential oils extracted from aerial parts of *A. millefolium* L., *M. longifolia* L., and *T. serpyllum* L. These species had variable volatile oil content (0.31% for *A. millefolium* L., 0.90% for *M. longifolia* L., and 0.43% for *T. serpyllun* L). The most abundant compounds of *A. millefolium* L., *M. longifolia* L., and *T. serpyllum* L. volatile oils were chamazulene (49.72%), pulegone (36.20%), and thymol (42.11%), respectively.

All three volatile oils demonstrated antioxidant and antimicrobial properties, suggesting their potential use in pharmaceutic, cosmetic, and food industries. *T. serpyllum* L. essential oil exerted the highest free radical scavenging activity, inhibiting the DPPH free radical by 98.19% and presenting a mean value of 1.82 mmol TE/mL EO in the ABTS test. *A. millefolium* L. essential oil was the most active in the FRAP assay, with a mean value of 66.70 μmol TE/mL EO. From the three types of plants harvested in the mountainous areas of Sibiu County, Romania, *T. serpyllum* L. produced an EO which exhibited strong antibacterial activity, rivaling standard antibiotics, on all tested bacteria, with the exception of *S. aureus* and *K. pneumoniae*. The maximum diameter of the inhibition zone was 37 mm for *B. subtilis*. The EO from *M. longifolia* L. showed resistance to intermediate action on the bacteria, being the better performer against *S. aureus* (ZOI = 15.33) and *S. typhi* (ZOI = 15 mm). The EO from *A. millefolium* L. demonstrated the weakest inhibitory effect, but with an action comparable to the other EOs on *S. typhi*. From the pathogenic bacteria, *S. typhi* showed the most sensitivity, whereas *K. pneumoniae* was resistant to all EOs.

A normal diet supplemented with *A. millefolium* L., *M. longifolia* L., and *T. serpyllum* L. volatile oils leads to a dose-dependent decrease in the viability of both larvae and pupae of *Drosophila melanogaster*. Moreover, morphological deformities were observed at higher concentrations. However, further studies are needed to elucidate which of the volatile oil components are responsible for the therapeutic and toxic effects and whether the effect occurs as a result of synergistic or antagonistic actions. Although in vitro studies suggest that volatile oils have antioxidant and antimicrobial properties, particular attention should be paid to toxic effects. Further in vivo studies are needed to elucidate their benefits and risks in humans before introducing them in therapy.

## Figures and Tables

**Table 1:** Extraction yields and organoleptic properties of volatile oils.

Plant Part	Mean Value of Extraction Yield ± Standard Deviation (%*v*/*w*)	Organoleptic Properties
*Achillea millefolium* L. aerial part	0.31 ± 0.04	dark blue liquid with a characteristic strong smell
*Mentha longifolia* L. aerial part	0.90 ± 0.65	yellowish liquid with a characteristic strong smell
*Thymus serpyllum* L. aerial part	0.43 ± 0.23	clear, yellow liquid with a characteristic strong smell

Where, *v*—volume in mL; *w*—dry weight in g.

**Table 2:** Chemical composition of *Achillea millefolium* L. volatile oil.

No.	Compound	RT	% Area (Mean Value ± Standard Deviation)
1	ɑ-pinene	12.675	1.09 ± 0.11
2	**sabinene**	14.101	**4.24 ± 0.14**
3	**β-pinene**	14.244	**7.00 ± 0.21**
4	β-mircene	14.487	0.62 ± 0.10
5	1,3,8-p-menthatriene	16.022	1.35 ± 0.12
6	**eucalyptol (1,8-cineole)**	16.182	**9.64 ± 0.18**
7	γ-terpinene	16.898	1.07 ± 0.20
8	trans-chrysanthemol	20.036	1.19 ± 0.15
9	camphor	20.253	1.01 ± 0.34
10	pinocarvone	20.747	0.46 ± 0.14
11	terpinen-4-ol	21.090	1.68 ± 0.15
12	ɑ-terpineol	21.580	2.79 ± 0.17
13	cis-myrtanol	23.231	3.83 ± 0.15
14	thymol	24.610	1.50 ± 0.10
15	**caryophyllene**	27.259	**4.12 ± 0.11**
16	humulene	28.256	0.50 ± 0.05
17	**germacrene D**	28.911	**4.69 ± 0.11**
18	nerolidol	30.645	0.58 ± 0.12
19	caryophyllene oxide	31.802	2.84 ± 0.12
20	**chamazulene**	35.500	**49.72 ± 0.24**
Total			99.92
Unidentified volatile constituents			0.08

RT: retention time; the text in bold highlights the oil’s major constituents, with a concentration exceeding 4%.

**Table 3:** Chemical composition of *Mentha longifolia* L. volatile oil.

No.	Compound	RT	% Area (Mean Value ± Standard Deviation)
1	ɑ-pinene	12.675	0.22 ± 0.04
2	β-phellandrene	14.093	0.17 ± 0.06
3	β-pinene	14.257	0.31 ± 0.12
4	β-myrcene	14.487	0.30 ± 0.11
5	1,3,8-p-menthatriene	16.022	1.00 ± 0.31
6	eucalyptol (1,8-cineole)	16.182	1.48 ± 0.09
7	γ-terpinene	16.898	0.14 ± 0.08
8	**cis-menthone**	20.427	**17.89 ± 0.32**
9	**isomenthone**	20.669	**4.52 ± 0.29**
10	trans-menthone	21.203	0.47 ± 0.13
11	ɑ-terpineol	21.588	0.24 ± 0.07
12	**pulegone**	23.010	**36.20 ± 0.34**
13	ascaridole	23.292	3.61 ± 0.15
14	**piperitone oxide**	23.470	**9.21 ± 0.18**
15	dihydroedulan II	23.916	0.67 ± 0.12
16	thymol	24.285	0.84 ± 0.12
17	**2,6-dimethyl hydroquinone**	26.570	**4.10 ± 0.22**
18	caryophyllene	27.272	**9.56 ± 0.20**
19	humulene	28.256	0.36 ± 0.04
20	**germacrene D**	28.919	**5.29 ± 0.15**
21	epizonarene	29.626	0.23 ± 0.06
22	caryophyllene oxide	31.807	2.49 ± 0.11
Total			99.33
Unidentified volatile constituents			0.67

RT: retention time; the text in bold highlights the oil’s major constituents, with a concentration exceeding 4%.

**Table 4:** Chemical composition of *Thymus serpyllum* L. volatile oil.

No.	Compound	RT	% Area (Mean Value ± Standard Deviation)
1	2-thujene	12.411	0.73 ± 0.12
2	ɑ-pinene	12.671	0.48 ± 0.13
3	camphene	13.312	0.29 ± 0.06
4	β-pinene	14.253	0.17 ± 0.07
5	1-octen-3-ol	14.583	0.59 ± 0.06
6	β-phellandrene	15.203	0.38 ± 0.10
7	terpinolene	15.541	1.46 ± 0.12
8	**p-cymene**	16.065	**16.34 ± 0.21**
9	eucalyptol (1,8-cineole)	16.178	0.30 ± 0.06
10	**γ-terpinene**	16.937	**10.97 ± 0.11**
11	linalool	18.315	0.19 ± 0.07
12	endo-borneol	20.856	0.27 ± 0.04
13	terpinen-4-ol	21.086	0.41 ± 0.09
14	**methyl thymol**	22.495	**4.45 ± 0.11**
15	**methyl carvacrol**	22.520	**6.03 ± 0.10**
16	**thymol**	24.367	**42.11 ± 0.39**
17	**carvacrol**	24.701	**8.71 ± 0.20**
18	caryophyllene	27.259	3.15 ± 0.12
19	β-bisabolene	29.236	1.73 ± 0.16
20	τ-cadinol	29.630	0.30 ± 0.06
21	caryophyllene oxide	31.802	0.55 ± 0.10
Total			99.61
Unidentified volatile constituents			0.39

RT: retention time; the text in bold highlights the oil’s major constituents, with a concentration exceeding 4%.

**Table 5:** Antioxidant potential of *Achillea millefolium* L., *Mentha longifolia* L., and *Thymus serpyllum* L.

Sample	Assay		
	RSA (%)	ABTS (mmol TE/mL EO)	FRAP (μmol TE/mL EO)
*A. millefolium* L.	87.87 ± 0.34 ^a^	0.83 ± 0.60 ^a^	66.70 ± 0.54 ^a^
*M. longifolia* L.	96.13 ± 0.46 ^b^	0.04 ± 0.01 ^a^	18.10 ± 0.45 ^a^
*T. serpyllum* L.	98.19 ± 0.57 ^c^	1.82 ± 0.07 ^a^	29.84 ± 0.51 ^a^
Ascorbic acid	100 ± 0.42 ^d^ *	21.45 ± 1.53 ^b^ mmol TE/g ascorbic acid	20.07 ± 1.09 ^b^ mmol TE/g ascorbic acid

RSA: DPPH• radical scavenging activity; ABTS: ABTS•+ radical cation scavenging assay; FRAP: ferric-reducing antioxidant power; TE: Trolox equivalent; EO: essential oil; *: ascorbic acid solution 1 mg/mL. Experiments were performed in triplicate (*n* = 3) and results were expressed as the mean ± standard deviation (SD). Values from the same column followed by different letters are significantly different (*p* < 0.05).

## Data Availability

The original contributions presented in this study are included in the article/Supplementary Materials. Further inquiries can be directed to the corresponding author(s).

## Supplementary Materials

The following supporting information can be downloaded at https://www.mdpi.com/article/10.3390/ph19081293/s1, Figure S1: GC-MS chromatogram of *Achillea millefolium* L. essential oil; Figure S2: GC-MS chromatogram of *Mentha longifolia* L. essential oil; Figure S3: GC-MS chromatogram of *Thymus serpyllum* L. essential oil.
