# Supplementary material for: Biological and Phytochemical Profile of Volatile Oils from Romanian Mountain Flora
Source: Pharmaceuticals (Basel). 2026 Aug 15;19(8):1293. doi: 10.3390/ph19081293 (PMC13517077; doi:10.3390/ph19081293)
Supplement: Supplementary file 1 [file pharmaceuticals-19-01293-s001.zip › pharmaceuticals-4472472-supplementary.pdf]

SUPPLEMENTARY MATERIAL

Biological and Phytochemical Profile of Volatile Oils from Romanian Mountain Flora

Lidia-Ioana Virchea [1], Maria Totan [1,*], Cecilia Georgescu [2,*], Adina Frum [1], Endre Máthé [3,4], Monica Mironescu [2], Bence Pecsenye [3], Robert Nagy [3], Oana Viorica Danci [5], Maria-Lucia Mureșan [1], Nastaca Alina Palade [1] and Felicia-Gabriela Gligor [1]

[1] Faculty of Medicine, "Lucian Blaga" University of Sibiu, Lucian Blaga Str. 2A, 550169 Sibiu, Romania; lidia.virchea@ulbsibiu.ro (L.-I.V.); adina.frum@ulbsibiu.ro (A.F.); maria.muresan@ulbsibiu.ro (M.-L.M.); nastacaalina.coman@ulbsibiu.ro (N.A.P.); felicia.gligor@ulbsibiu.ro (F.-G.G.)

[2] Faculty of Agriculture Sciences, Food Industry and Environmental Protection, "Lucian Blaga" University of Sibiu, Dr. Ion Rațiu Str. 7-9, 550012 Sibiu, Romania; monica.mironescu@ulbsibiu.ro

[3] Institute of Nutrition Science, Faculty of Agricultural and Food Sciences and Environmental Management, University of Debrecen, Böszörményi Str. 128, 4032 Debrecen, Hungary; endre.mathe@agr.unideb.hu (E.M.); pecsenye.bence@agr.unideb.hu (B.P.); nagy.robert@agr.unideb.hu (R.N.)

[4] Department of Life Sciences, Faculty of Medicine, Vasile Goldis Western University of Arad, L. Rebreanu Str. 86, 310414 Arad, Romania

[5] Faculty of Sciences, "Lucian Blaga" University of Sibiu, Dr. Ion Rațiu Str. 5-7, 550012 Sibiu, Romania; oana.danci@ulbsibiu.ro

* Correspondence: maria.totan@ulbsibiu.ro (M.T.); cecilia.georgescu@ulbsibiu.ro (C.G.)

Supplementary Figures

Figure S1. GC-MS chromatogram of *Achillea millefolium* L. essential oil;

Figure S2. GC-MS chromatogram of *Mentha longifolia* L. essential oil;

Figure S3. GC-MS chromatogram of *Thymus serpyllum* L. essential oil.

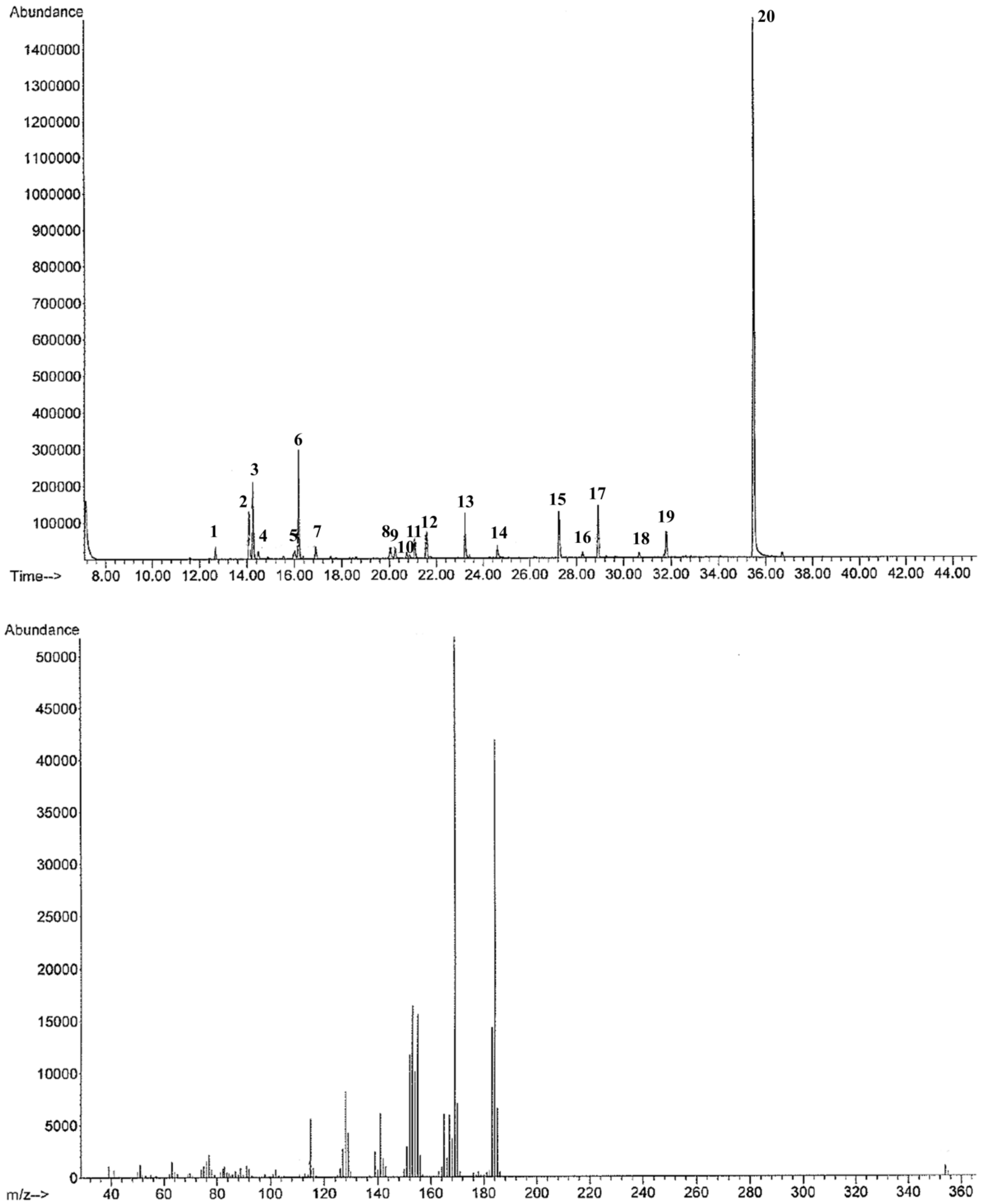

Figure S1. GC-MS chromatogram of *Achillea millefolium* L. essential oil.

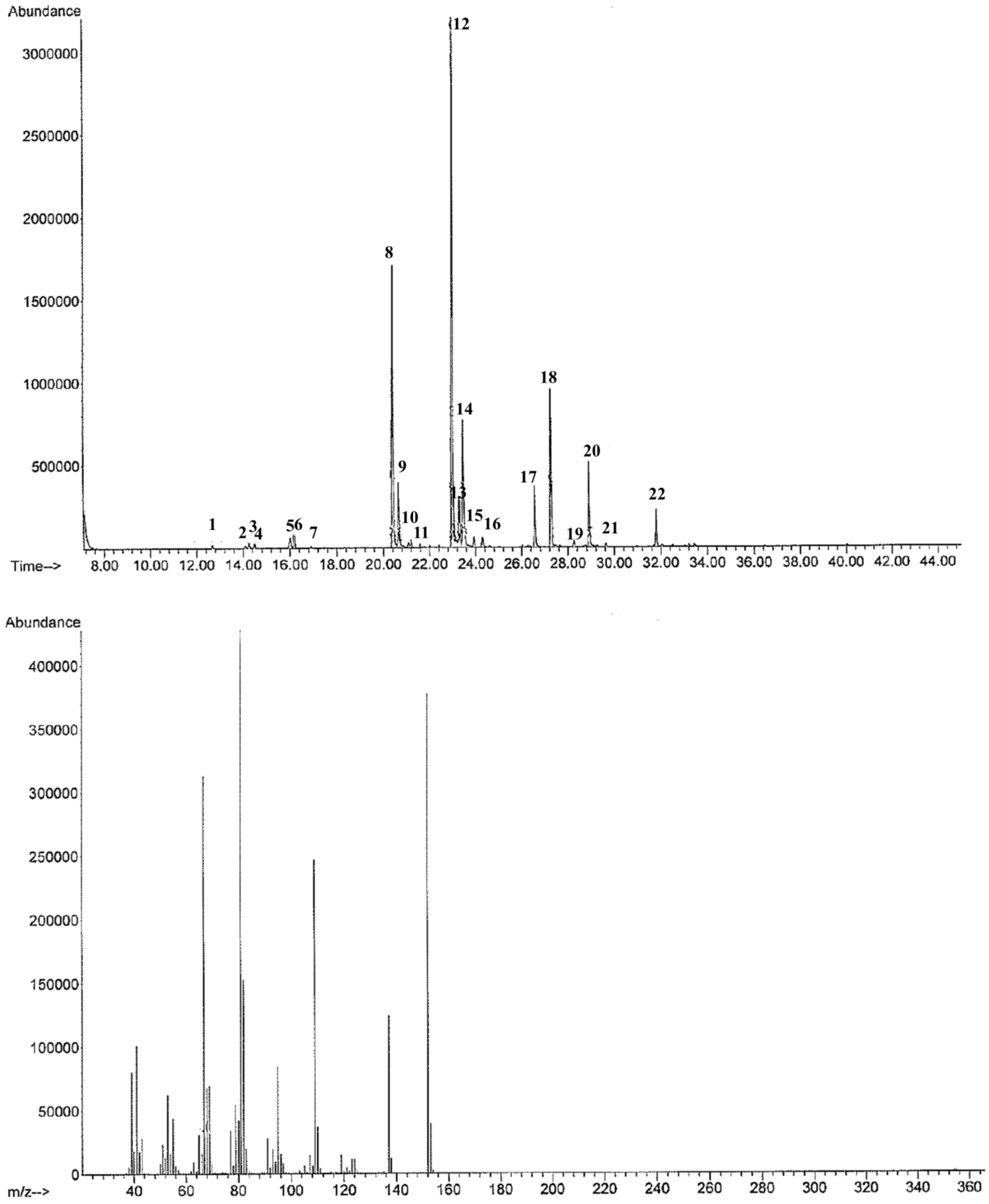

Figure S2. GC-MS chromatogram of *Mentha longifolia* L. essential oil.

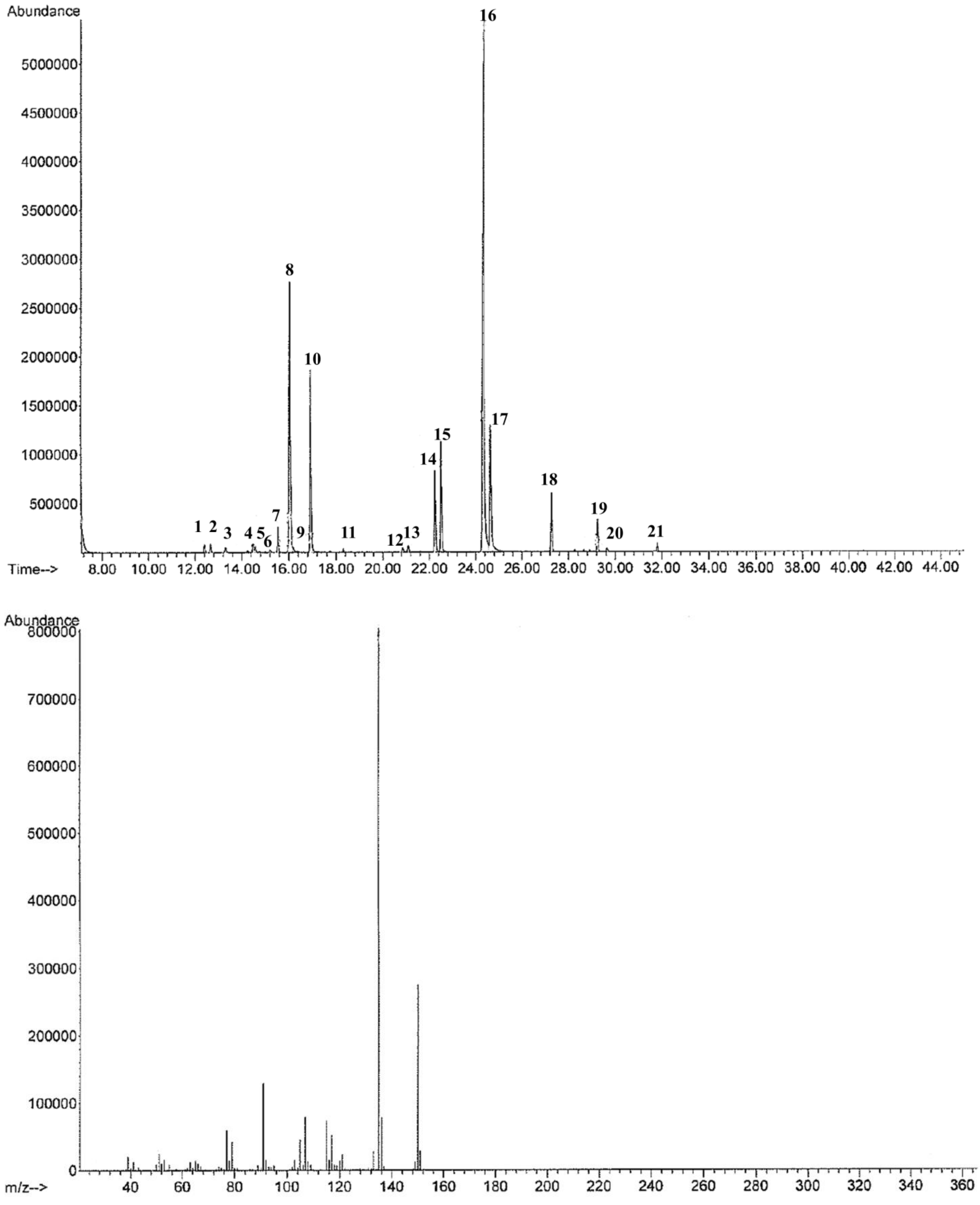

Figure S3. GC-MS chromatogram of *Thymus serpyllum* L. essential oil.
